# From Activity Screening to Quality Control: UHPLC-MS/MS Analysis of Anti-Inflammatory Cyclodipeptides in *Pinellia ternata*

**DOI:** 10.3390/molecules31081322

**Published:** 2026-04-17

**Authors:** Yue Wang, Yunyun Luo, Jingjing Gan, Li Wang, Cuifen Fang, Linlin Zhang, Cheng Zhen, Bilian Chen

**Affiliations:** 1 School of Pharmaceutical Sciences, Zhejiang Chinese Medical University, Hangzhou 310053, China; wangyueae@163.com (Y.W.); 19560482816@163.com (L.W.); 2 NMPA Key Laboratory of Quality Evaluation of Traditional Chinese Medicine (Traditional Chinese Patent Medicine), Zhejiang Institute for Food and Drug Control, Hangzhou 310052, China; fcf0507@126.com (C.F.); amazing1212@163.com (L.Z.); joeff30@163.com (C.Z.); 3 Zhejiang Key Laboratory of Biopharmaceutical Contact Materials, Hangzhou 310052, China; 4 Key Laboratory of Quality and Safety of Functional Food, State Administration for Market Regulation, Hangzhou 310052, China; 5 School of Pharmacy, Hangzhou Normal University, Hangzhou 311121, China; 18577572565@163.com

**Keywords:** *Pinellia ternata*, cyclodipeptide, anti-inflammatory activity, material basis, quality control

## Abstract

This study investigated the anti-inflammatory material basis and quality control of *Pinellia ternata* (*P. ternata*) to provide a modern scientific interpretation for its therapeutic properties. First, ultra-high-performance liquid chromatography coupled with quadrupole time-of-flight tandem mass spectrometry (UHPLC-Q-TOF-MS/MS) analysis was used to analyze different polar fractions of *P. ternata*, resulting in the identification of 79 compounds. Next, an in vitro evaluation using an LPS-induced RAW264.7 cell inflammation model revealed that the ethyl acetate fraction exhibited the most significant inhibition of nitric oxide (NO) production. Three cyclodipeptides, cyclo (Pro-Leu), cyclo (Phe-Pro), and cyclo (Leu-Phe), which displayed notable differences from other fractions, were subsequently screened. Molecular docking studies showed binding free energies below −5 kcal/mol with inducible nitric oxide synthase (iNOS), indicating potential anti-inflammatory targeting properties. Cellular experiments further confirmed that the reduction in NO production induced by these cyclodipeptides ranged from 11.03% to 40.38%. To enable their simultaneous quantification, a method based on ultra-high-performance liquid chromatography–triple quadrupole tandem mass spectrometry (UHPLC-QQQ-MS/MS) in the multiple reaction monitoring (MRM) mode was established, meeting all analytical validation criteria. Application of this method to *P. ternata* samples from different origins and growth conditions demonstrated that the contents of these cyclodipeptides were significantly influenced by both the origin and cultivation method. In conclusion, this study preliminarily identifies cyclodipeptides as an important anti-inflammatory material basis of *P. ternata*, and the established quantitative method provides methodological support and data for constructing its quality evaluation system.

## 1. Introduction

The global prevalence of chronic inflammatory diseases represents a major public health challenge of the 21st century [1,2]. These conditions are intricately linked to metabolic syndrome, neurodegenerative diseases, and various malignant tumors, placing a substantial burden on healthcare systems and socioeconomic development [3,4,5]. Consequently, modulating excessive and persistent inflammatory responses has become a primary therapeutic objective. However, the long-term administration of conventional anti-inflammatory agents, such as non-steroidal anti-inflammatory drugs (NSAIDs) and glucocorticoids, is frequently associated with severe adverse effects, including gastrointestinal damage, increased cardiovascular risks, and systemic immunosuppression. Furthermore, the development of drug resistance during prolonged treatment increasingly compromises their clinical efficacy [6,7,8,9,10,11]. As a result, the discovery of novel, highly effective anti-inflammatory lead compounds from traditional medicinal plants has become a critical strategy [12,13,14]. Natural products enable multi-target modulation of inflammatory pathways; for example, flavonoids, diterpenoids, and polyphenols are known to inhibit key pathways such as NF-κB, JAK-STAT, and NLRP3, offering promising avenues for the development of safer anti-inflammatory therapeutics [15,16,17].

NO plays a dual role in inflammation. Under physiological conditions, constitutive nitric oxide synthase (cNOS) generates trace amounts of NO, which exerts anti-inflammatory and protective effects. However, in inflammatory states, inducible nitric oxide synthase (iNOS) is upregulated, resulting in the excessive production of NO. This excess NO can be converted into potent cytotoxic species, such as peroxynitrite, causing tissue damage and amplifying the inflammatory cascade, thereby establishing NO as a critical inflammatory mediator [18,19]. Consequently, suppressing iNOS overexpression represents a vital therapeutic strategy for managing chronic inflammation. In this context, the traditional Chinese medicinal herb *P. ternata* presents significant research value. Widely used in the clinical practice of Traditional Chinese Medicine (TCM), it is primarily employed to alleviate inflammation-related symptoms attributed to pathological factors such as phlegm-dampness, blood stasis, and qi stagnation [20]. While contemporary research has confirmed the anti-inflammatory properties of *P. ternata*, existing studies have primarily focused on the assessment of total extracts [21,22]. Although reports suggest that its extracts may exert effects by modulating endoplasmic reticulum stress and inhibiting the TLR4/NF-κB pathway and NLRP3 inflammasome activation, the specific anti-inflammatory constituents and their modes of action have yet to be fully clarified [23,24]. Furthermore, the yield and quality of *P. ternata* are significantly influenced by environmental and agronomic factors, as well as the complexity of its chemical components, posing challenges to quality consistency [25,26]. This research gap impedes the establishment of quality control standards based on anti-inflammatory efficacy, making it difficult to guarantee the consistent quality and efficacy of *P. ternata* products on the market. Therefore, there is an urgent need to establish quality evaluation methods for *P. ternata* to assess market quality.

To address the aforementioned issues, this study aimed to systematically elucidate the bioactive chemical basis underlying the anti-inflammatory effects of *P*. *ternata* and establish key technologies for its quality control based on the pivotal bioactive components. Initially, untargeted chemical profiling of different polar fractions of *P. ternata* was performed using UHPLC-Q-TOF-MS/MS. Bioactivity-guided screening, utilizing an LPS-induced RAW264.7 macrophage inflammation model, identified the most active fraction. Subsequently, through the integration of molecular docking simulations, three compounds exhibiting significant differences from other fractions were screened from this active fraction and structurally characterized as cyclodipeptides, namely cyclo(Pro-Leu), cyclo(Phe-Pro), and cyclo(Leu-Phe). The anti-inflammatory activities of these cyclodipeptides were further validated through in vitro assays using chemically synthesized pure compounds. Finally, a reliable UHPLC-MS/MS (MRM) method was established for the simultaneous quantification of these three cyclodipeptides, providing methodological support for the quality evaluation and further investigation of *P. ternata*.

## 2. Results

### 2.1. Characterization of Constituents in Different Polar Fractions Based on UHPLC-Q-TOF-MS/MS

**Figure 1:**
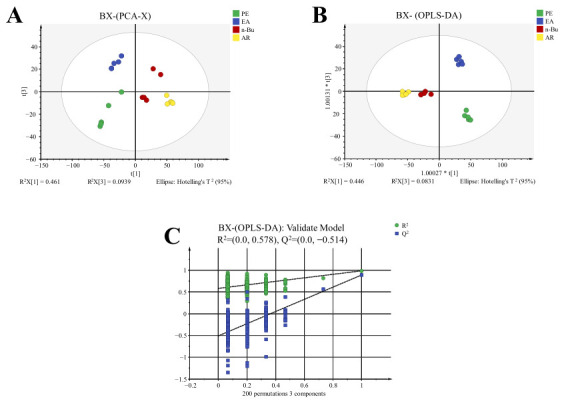
(**A**) PCA score plot of different polar fractions of *P. ternata*. (**B**) OPLS-DA score plot of different polar fractions of *P. ternata*. (**C**) Permutation test plot for the OPLS-DA model. Asterisks in axis labels (e.g., 1.00027 * t[1]) denote SIMCA scaling factors.

**Figure A1:**
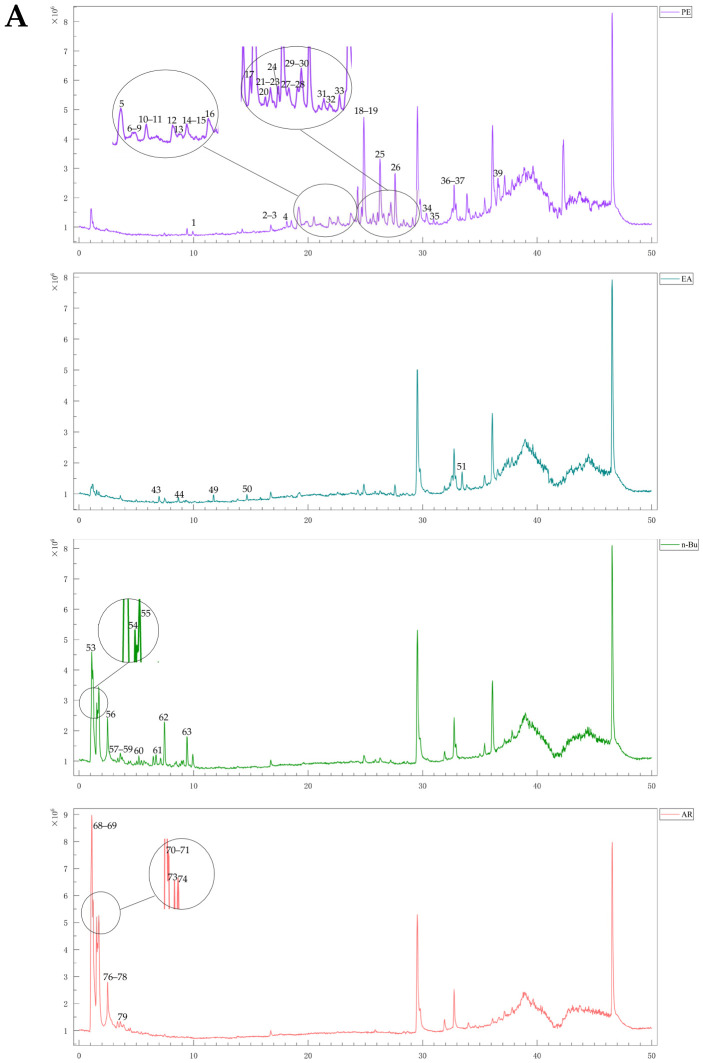

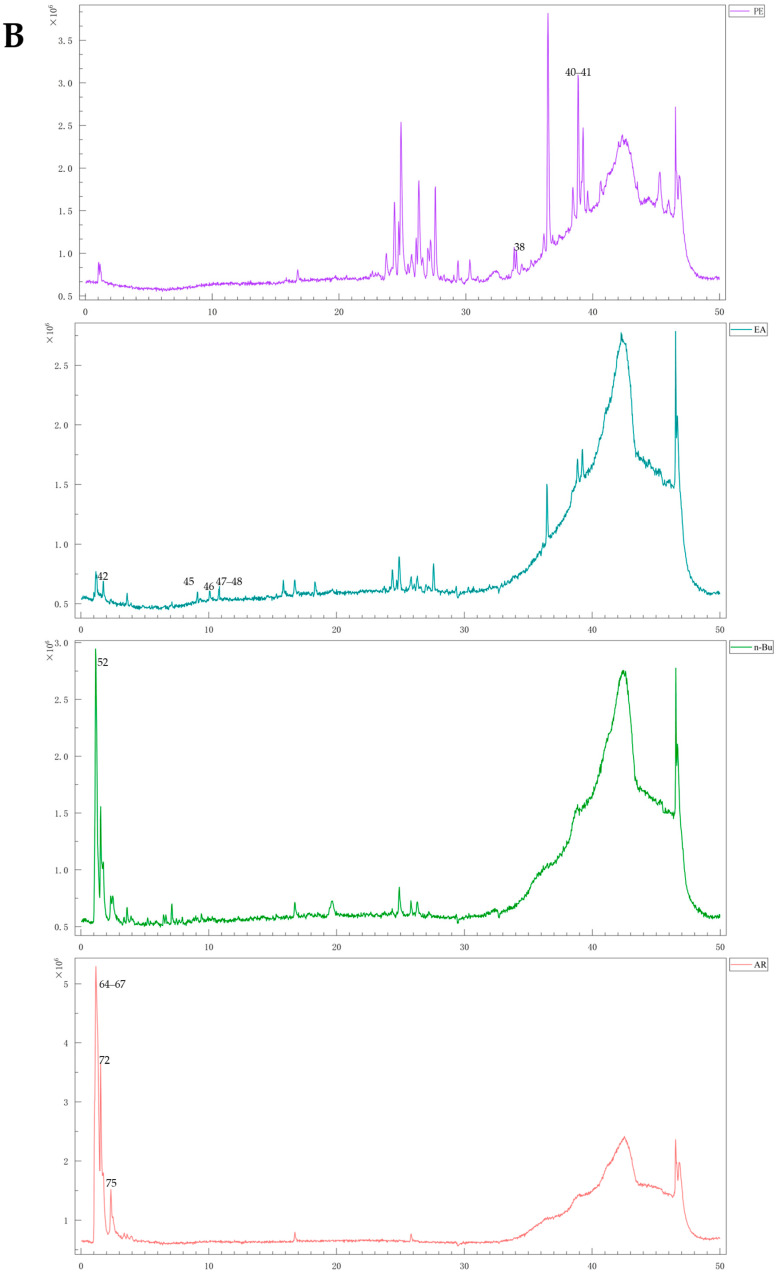
(**A**) Plot of the TIC in positive ion mode. (**B**) Plot of the TIC in negative ion mode.

A systematic chemical characterization of distinct extract fractions of *P. ternata*, specifically the petroleum ether (PE), ethyl acetate (EA), n-butanol (n-Bu), and aqueous residue (AR) fractions, was conducted via UHPLC-Q-TOF-MS/MS under dual-ionization (positive/negative) mode, with the corresponding total ion chromatograms provided in Appendix Figure A1. Principal component analysis (PCA) and orthogonal partial least squares-discriminant analysis (OPLS-DA) were employed to discriminate the chemical profiles among these polar fractions. PCA revealed distinct separation among the fractions (Figure 1A). Further OPLS-DA analysis (Figure 1B) showed clear classification trends and significant clustering characteristics. The model demonstrated a cumulative explanation capability of *R*^2^*X* = 0.759 and *R*^2^*Y* = 0.985, with a predictive parameter Q^2^ = 0.918. Both *R*^2^ and *Q*^2^ values exceeded 0.5, and all samples fell within the 95% confidence interval, indicating a stable, accurate, and reliable model. To validate the model’s fitness, a 200-permutation test was conducted (Figure 1C). The resulting regression line exhibited a negative intercept on the y-axis, confirming the absence of overfitting and supporting the model’s reliability. Based on the OPLS-DA model, differential compounds were screened using a variable importance in projection (VIP) threshold > 1.0 and significance testing (*p* < 0.05). By comparing retention times with reference standards and consulting literature data, a total of 79 compounds were identified (Table 1). The MS/MS spectra of all identified compounds are provided in the Supplementary Materials, along with possible fragmentation pathways for some compounds. It is important to note that for compounds identified without reference standards, stereochemical configurations were inferred by matching experimental MS/MS data with literature values. While these configurations represent the best fit for the available data, the presence of alternative stereoisomers cannot be definitively ruled out.

### 2.2. Bioactivity Evaluation of Different Polar Fractions

**Figure 2:**
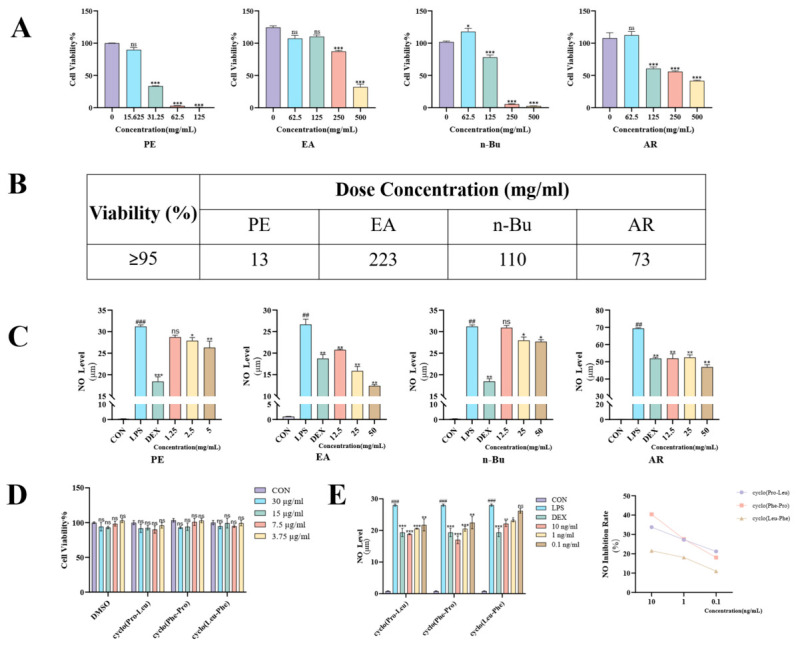
(**A**) Cell viability assay of different polar fractions on LPS-activated RAW 264.7 cells. (**B**) The safe concentration (cell viability ≥ 95%) for each fraction. (**C**) Effects of different polar fractions on NO inhibition in LPS-activated RAW 264.7 cells. (**D**) Cell viability assay of the three cyclodipeptides on LPS-activated RAW 264.7 cells. (**E**) Effects of the three cyclodipeptides on NO inhibition in LPS-activated RAW 264.7 cells, as determined by the Griess reagent method; data are presented as the mean ± SEM (*n* = 3). # *p* < 0.05, ## *p* < 0.01, ### *p* < 0.001 versus the control group; * *p* < 0.05, ** *p* < 0.01, *** *p* < 0.001 versus the LPS-treated group; ns, not significant (*p* > 0.05).

The CCK-8 assay indicated that cell viability remained above 95% (no significant cytotoxicity) when the concentrations (all extract concentrations in this study are expressed and presented as raw herb equivalents) of the PE, EA, n-Bu, and AR fractions were below 13, 223, 110, and 73 mg/mL, respectively (Figure 2A,B). The anti-inflammatory activity of each fraction was further evaluated by measuring the inhibition of NO production using the Griess assay. All fractions suppressed NO generation to varying degrees (Figure 2C). Notably, the EA fraction exhibited the most potent activity, demonstrating a strong, dose-dependent inhibition of NO within the concentration range of 12.5–50 mg/mL. While the AR fraction also inhibited NO production, its effect was weaker and less dose-dependent compared to the EA fraction within the same range. Due to its inherent cytotoxicity at effective concentrations, the PE fraction did not show significant NO inhibition below its safety threshold. The n-Bu fraction displayed no marked anti-inflammatory activity. These results suggest that the EA fraction is enriched with potent anti-inflammatory constituents, highlighting its potential as a valuable source for anti-inflammatory lead compounds from *P. ternata*.

### 2.3. Prediction of Anti-Inflammatory Active Components in the Ethyl Acetate Fraction

**Figure 3:**
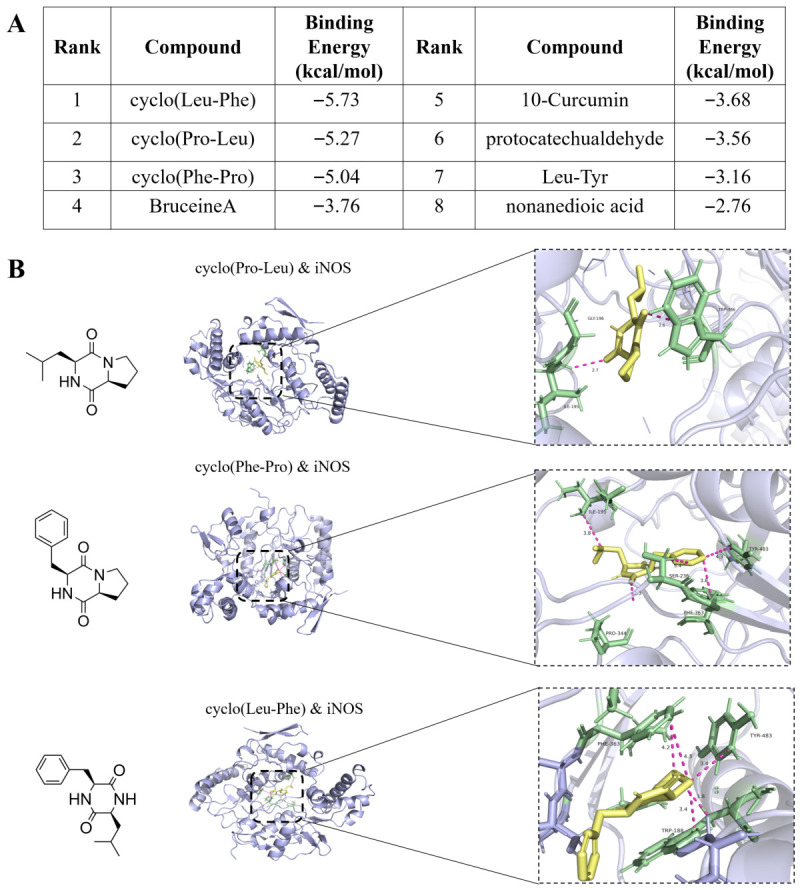
(**A**) Prediction of the binding energy between components from the EA fraction and iNOS. (**B**) Molecular docking simulation of three cyclodipeptides with iNOS. Key binding site residues are shown as green sticks, the ligands are shown as yellow sticks, and pink dashed lines represent hydrogen bonds with distances indicated in Ångströms (Å).

Eight known differential small-molecule compounds extracted from the EA fraction were used as ligands, with inducible nitric oxide synthase (iNOS)—a key protein directly catalyzing NO production in the inflammatory response—serving as the receptor protein for molecular docking. Using the AutoDockTools software platform (version 1.5.7), systematic molecular docking simulations between the ligands and the receptor were conducted, and the binding free energy (ΔG) of each complex system was calculated and ranked (Figure 3A). Generally, a more negative binding free energy (i.e., a larger absolute value) indicates greater stability of the ligand-receptor binding and a higher likelihood that the molecule represents a potential active component. As shown in Figure 3, among all tested small molecules, the three cyclodipeptides exhibited binding free energies (ΔG) with iNOS protein below −5 kcal/mol, demonstrating strong binding affinity (Figure 3B). This suggests that these cyclodipeptides may possess significant potential to inhibit iNOS activity.

### 2.4. In Vitro Activity Validation of Cyclodipeptide Components

Cell viability assays indicated that cyclo(Pro-Leu), cyclo(Phe-Pro), and cyclo(Leu-Phe) exhibited no cytotoxicity toward RAW 264.7 macrophages at concentrations below 30 μg/mL (Figure 2D). Evaluation using the Griess reagent method revealed that these three cyclodipeptides inhibited nitric oxide (NO) production in an LPS-induced inflammation model (Figure 2E). Among them, cyclo(Pro-Leu) and cyclo(Phe-Pro) demonstrated a significant dose-dependent inhibitory effect on NO regulation, while cyclo(Leu-Phe) also exhibited a weak downregulatory capacity. Within the concentration range of 0.1–10 ng/mL, the NO inhibition rates ranged from 11.03% to 40.38%. These results indicate that the cyclodipeptides identified from the EA fraction constitute the key material basis for its anti-inflammatory effects.

### 2.5. Establishment and Analysis of the Quantitative Method for Active Cyclodipeptides

#### 2.5.1. Specificity

**Figure 4:**
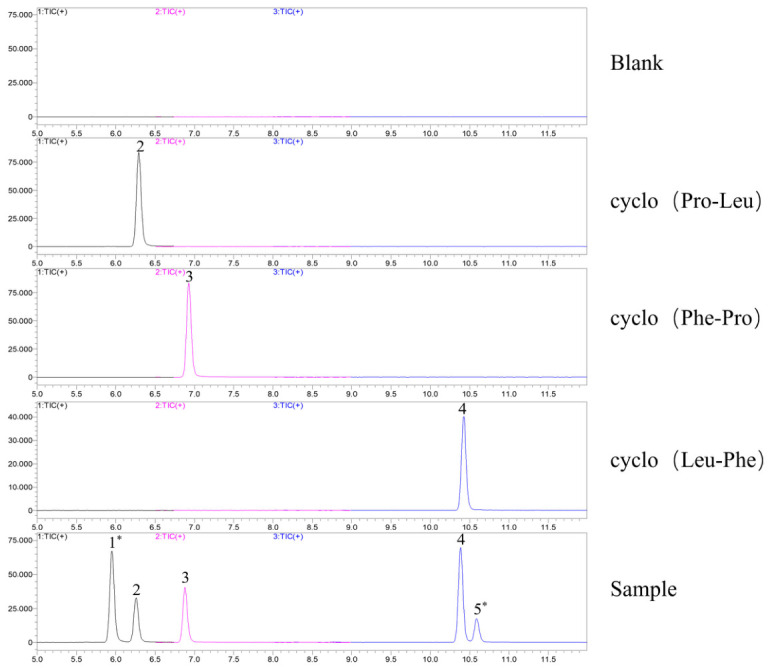
Comparative UHPLC-MS/MS (MRM) chromatograms of reference standards and a *P. ternata* sample for the cyclodipeptides cyclo(Pro-Leu), cyclo(Phe-Pro), and cyclo(Leu-Phe). Peaks 2, 3, and 4 were identified by co-elution with authentic standards. Peaks 1* and 5*, detected using identical compound-specific MRM transitions, did not match the retention times of the available standards. These peaks are proposed to be isomeric or structurally related components sharing the targeted mass spectrometric signature.

From the MRM chromatograms of the standard solutions of the three cyclodipeptides and the blank sample (Figure 4), no interfering peaks were observed at the respective retention times of each target compound, demonstrating good specificity of the developed method.

#### 2.5.2. Linearity and Limit of Detection

The calibration curves for the three cyclodipeptides all demonstrated good linearity across their respective concentration ranges, with coefficients of determination (R^2^) exceeding 0.99 (Table 2), confirming a well-fitted linear relationship for each compound. The limit of detection (LOD) and limit of quantitation (LOQ) were determined based on signal-to-noise (S/N) ratios. The LOD was defined as the concentration yielding an S/N ratio of 3, while the LOQ was established as the lowest concentration on the calibration curve exhibiting an S/N ratio greater than 10, thereby ensuring reliable quantification.

#### 2.5.3. Matrix Effect

The matrix effects for the three cyclodipeptides ranged from 89% to 97% (Table 3), indicating minimal interference from the sample matrix.

#### 2.5.4. Precision

The precision of the method, expressed as the relative standard deviation (RSD), was between 0.35% and 0.69% for the three cyclodipeptides (Table 4), demonstrating excellent instrumental precision.

#### 2.5.5. Stability

The stability of the analytes in the sample solution was confirmed over 24 h, with RSD values ranging from 0.37% to 0.81% (Table 5).

#### 2.5.6. Repeatability

The method showed good repeatability, as evidenced by RSDs of 0.36% to 1.53% for the three cyclodipeptides across six independently prepared sample solutions (Table 6).

#### 2.5.7. Recovery

The mean recovery rates for the three cyclodipeptides ranged from 84% to 110% (Table 7), confirming the good accuracy of the method. This validated method is thus suitable for the quantitative determination of these three cyclodipeptides in *P. ternata*.

#### 2.5.8. Quantification of Cyclodipeptides in P. *ternata* from Different Origins and Sources

**Figure 5:**
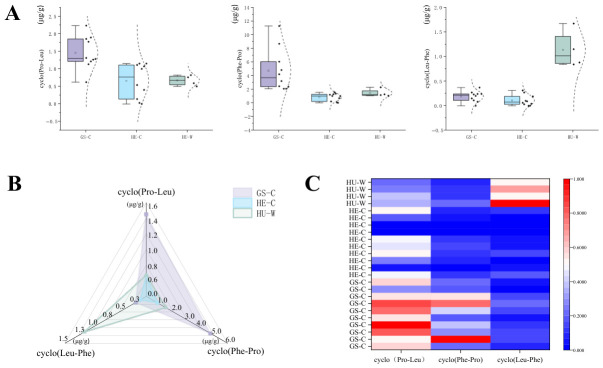
(**A**) Box plots, (**B**) radar chart, and (**C**) cluster analysis heatmap showing the content differences in *P. ternata* from different geographical origins and sources. GS, HE, and HU indicate samples from Gansu Province, Hebei Province, and Hubei Province of China, respectively; W represents wild *P. ternata*, and C represents cultivated *P. ternata*.

The contents of the three cyclodipeptides in *P. ternata* samples from different geographical origins and sources were compared using box plots, radar charts, and clustered heatmap analysis (Figure 5A–C). The results revealed significant variation in cyclodipeptide composition across samples. When grouped by geographical origin, samples from the GS region showed notably higher median levels of both cyclo(Pro-Leu) and cyclo(Phe-Pro) compared to other groups, suggesting a distinct chemotypic profile favorable for the accumulation of these compounds. Furthermore, when categorized by source, wild-collected samples contained substantially higher amounts of cyclo(Leu-Phe) than cultivated samples, underscoring the phytochemical distinctiveness of wild *P. ternata*. These findings provide a concrete basis for the quality assessment of *P. ternata* and offer valuable insights for the targeted utilization and development of material from specific origins and sources.

## 3. Discussion

Cyclopeptide natural products from plants, particularly major subclasses such as cyclodipeptides (also known as diketopiperazines) and cyclodepsipeptides, represent a significant source for drug discovery due to their remarkable chemical diversity and broad spectrum of bioactivities. Notably, over 40 cyclic peptide-based drugs are currently in clinical use [31,32,33,34]. In recent years, there has been a growing number of cyclopeptides (e.g., caripe 8 and cyclic peptide GG-8-6 analogues) isolated and characterized from medicinal plants. Their pronounced biological activities underscore the considerable potential of this class of compounds as promising lead candidates for novel therapeutics [35,36].

Cyclodipeptides, the simplest class of cyclic peptides found in nature, are structurally characterized by a stable six-membered ring formed from two amino acids linked via an amide bond. Owing to their highly rigid molecular conformation and favorable structural modifiability, cyclodipeptides exhibit broad application prospects in drug discovery and have become important targets for the design and functional validation of bioactive molecules [37]. These compounds not only demonstrate significant pharmacological value in various areas, including anticancer, neuroprotective, and cardioprotective effects, but also show potential protective benefits in the treatment of respiratory diseases [38]. In the quality assessment of medicinal plants, the quantitative analysis of cyclopeptides has been effectively employed as a dependable approach. For example, it has been utilized in the evaluation of herbs such as *Pseudostellaria heterophylla* [39]. This established approach provides a strong precedent for utilizing specific cyclodipeptides as objective chemical markers for quality evaluation. Notably, among the various cyclodipeptides identified in *P. ternata*, compounds such as cyclo(Leu-Pro) and cyclo(Phe-Pro) have emerged as promising candidates for this purpose due to their notable biological activities. Specifically, cyclo(Leu-Pro) acts as a scavenger of oxygen free radicals such as O_2_^−^• and OH•, while cyclo(Phe-Pro) exhibits even stronger inhibitory activity against OH• [40]. Moreover, the combined use of cyclo(Leu-Pro) and cyclo(Phe-Pro) displays significant synergistic antibacterial effects against pathogenic Salmonella [41]. These documented biological activities emphasize their potential functional significance and provide support for their selection as crucial chemical markers in the development of a quality control system tailored to *P. ternata*.

In the present study, cyclodipeptides identified from the medicinal plant *P. ternata*—namely cyclo(Pro-Leu), cyclo(Phe-Pro), and cyclo(Leu-Phe)—demonstrated significant NO-modulating activity. While cyclodipeptides themselves possess anti-inflammatory properties, potential synergistic effects with other compounds in the extract cannot be excluded. Such synergy may be an important factor contributing to its overall anti-inflammatory activity. Building upon these findings, this study proceeded to quantitatively analyze the cyclodipeptides. Using the established quantitative method, the contents of these three cyclodipeptides were determined in *P. ternata* samples from different geographical origins and sources, revealing distinct variation in their levels. Among samples grouped by origin, those from Gansu Province showed higher contents of cyclo(Pro-Leu) and cyclo(Phe-Pro). When grouped by source, wild samples contained markedly higher levels of cyclo(Leu-Phe) compared to cultivated ones. These results suggest that the growth environment of *P. ternata* influences its cyclodipeptide content. Since cyclodipeptides are likely synergistic contributors to the anti-inflammatory efficacy, samples from different origins and sources may vary in therapeutic outcome. In clinical practice, therefore, the geographical origin and source of *P. ternata* should be considered to ensure consistent medicinal effects. Beyond traditional morphological and macroscopic criteria, cyclodipeptide content can serve as an important reference index, aiding in the establishment of a more scientific and comprehensive quality evaluation system to guarantee the quality of this herbal medicine. Future research may further elucidate the biosynthetic pathways of cyclodipeptides during the growth of *P. ternata* and explore how growing conditions can be regulated to enhance their accumulation. Such work would provide a theoretical foundation for standardised cultivation and efficacy improvement of *P. ternata,* promote high-quality development of the related industry, and ultimately contribute further to human health.

## 4. Materials and Methods

### 4.1. Materials and Reagents

All samples (Table 8) were authenticated by Professor Zengxi Guo (Zhejiang Institute for Food and Drug Control).

Chromatographic-grade methanol and acetonitrile were purchased from Merck (Rahway, NJ, USA). Petroleum ether, ethyl acetate, n-butanol, methanol, and ethanol were obtained from Sinopharm Chemical Reagent Co., Ltd. (Shanghai, China). Deionized water was prepared using a Milli-Q integral water purification system (MilliporeSigma, Burlington, MA, USA). RAW 264.7 macrophages were acquired from the Cell Bank of the Committee on Type Culture Collection, Chinese Academy of Sciences (Shanghai, China). Fetal bovine serum and DMEM high-glucose medium were purchased from Shanghai Datherser Biological Technology Co., Ltd. (Shanghai, China). CCK-8 and NO assay kits were supplied by Shanghai Beyotime Biotechnology Co., Ltd. (Shanghai, China). Phosphate-buffered saline (PBS) was purchased from Zhejiang Senrui Biotechnology Co., Ltd. (Hangzhou, China) The reference standards cyclo(Pro-Leu) and cyclo(Phe-Pro) were purchased from Sen’er Biotechnology Co., Ltd. (Hefei, China); cyclo(Leu-Phe) was obtained from Yuanye Biotechnology Co., Ltd. (Shanghai, China), all with purities > 98%. The reference standards of succinate, uridine, adenosine, guanine, guanosine, tryptophan, methionine, and tyrosine were obtained from the National Institutes for Food and Drug Control (Beijing, China), all with purities > 90%.

### 4.2. Extraction of Different Polar Fractions from P. ternata

#### 4.2.1. Preparation of Crude Extracts for Cellular Administration

Powdered samples of *P. ternata* (S1–S24) were thoroughly mixed, and 200 g of the mixture was extracted with 9 volumes of 70% ethanol under reflux for 2 h. The extract was concentrated under reduced pressure using a rotary evaporator (BUCHI Labortechnik AG, Flawil, Switzerland) until no ethanol odor was detected. Sequential liquid–liquid partition was then performed. The concentrate was successively extracted with PE, EA, and water-saturated n-Bu. Each extraction was carried out twice with a solvent-to-solution ratio of 1:2 (*v*/*v*), and the respective organic layers were combined. The remaining aqueous phase was designated as the AR fraction. All fractions (PE, EA, n-Bu, and AR) were concentrated under reduced pressure, lyophilized, and then reconstituted in 5 mL of water to obtain stock solutions with a concentration of 40 g/mL. These stock solutions were stored at −80 °C for subsequent experiments.

Stock solutions of the three cyclodipeptides were prepared in 60% DMSO at a concentration of 1 mg/mL. Dexamethasone (DEX), (National Institutes for Food and Drug Control, Beijing, China) used as a positive control drug, was dissolved in DMSO to prepare a 1 mM stock solution. All stock solutions were stored at −80 °C for later use.

#### 4.2.2. Preparation of Different Polar Fractions for Q-TOF-MS/MS Component Analysis

Five batches of samples (S1, S2, S11, S12, S21) were selected. From each, 5 g of powder was processed according to the method described in Section 4.2.1. The resulting fractions were finally reconstituted with methanol in a 50 mL volumetric flask to obtain the test solutions for Q-TOF-MS/MS component analysis. Mass spectral data for positive and negative ionization modes were acquired through two independent sample injections. According to the method described above, each sample was prepared once to obtain five test solutions, which were then analyzed in positive and negative ion modes, resulting in a total of 10 acquisitions.

#### 4.2.3. Preparation of Test Solutions for Quantification of the Three Cyclodipeptides

Approximately 0.5 g of *P. ternata* powder was sieved through a No. 4 sieve and then accurately weighed into a 20 mL volumetric flask. An appropriate amount of extraction solvent (aqueous methanol, 30% *v*/*v*) was added, and the mixture was ultrasonically extracted for 30 min. The solution was subsequently diluted to the mark with the same solvent, mixed thoroughly to obtain the test solution.

For quantitative analysis, each sample was subjected to two independent sample preparations, each followed by a single LC-MS/MS injection. The final concentration was calculated as the mean of the two replicate measurements (*n* = 2).

### 4.3. Preparation of Standard Solutions and Working Solutions

An appropriate amount of each of the three cyclodipeptide reference standards was accurately weighed into separate volumetric flasks. The standards were dissolved and diluted to the mark with methanol to obtain individual stock solutions (1 μg/mL).

A series of working solutions at concentrations of 0.001, 0.01, 0.03, 0.06, and 0.125 μg/mL were prepared by appropriate dilution of the stock solutions and used to establish the calibration curves.

### 4.4. Cell Culture

RAW 264.7 murine macrophages were cultured in Dulbecco’s Modified Eagle Medium (DMEM), high glucose, supplemented with 10% fetal bovine serum, and maintained at 37 °C in a humidified incubator (Thermo Fisher Scientific Inc., Waltham, MA, USA) with 5% CO_2_.

### 4.5. Mass Spectrometric Analysis

#### 4.5.1. Qualitative MS Analysis

Qualitative analysis was conducted using an Agilent 1290 series UHPLC system interfaced with an Agilent 6545 Q-TOF mass spectrometer (Agilent Technologies, Santa Clara, CA, USA). The mass spectrometer was calibrated before each analysis using a standard tuning solution. Instrument control, data acquisition, and processing were performed with Agilent MassHunter Workstation Software (version B.08.00). An electrospray ionization (ESI) source was employed in Auto MS/MS mode to acquire data in both positive and negative polarities over a mass range of *m*/*z* 100–1500. The ESI conditions were optimized as follows: drying gas temperature, 320 °C at a flow of 8 L/min; nebulizer gas (N_2_) pressure, 35 psi; fragmentor voltage, 1000 V; sheath gas temperature, 350 °C at a flow of 11 L/min; capillary voltage, 3500 V. For collision-induced dissociation (CID) in MS/MS mode, collision energies of 20 and 40 eV were applied.

Chromatographic separation was achieved using an Agilent Extend-C18 column (2.1 × 150 mm, 1.8 μm) (Agilent Technologies, Santa Clara, CA, USA) maintained at 35 °C. The mobile phase consisted of (A) 0.1% formic acid in water and (B) acetonitrile. A linear gradient was applied from 5% to 95% B over 45 min at a constant flow rate of 0.3 mL/min. The injection volume was 1 μL.

To investigate the consistent presence of compounds in different samples, qualitative analysis was performed using UHPLC-Q-TOF(Agilent Technologies, Santa Clara, CA, USA) for the representative samples (S1, S2, S11, S12, S21) described in Section 4.2.2. By comparing the detection results of each sample, compounds stably detected in all samples were screened and recorded, and the mass spectrometric identification results of Sample S1 were used as the final dataset. In this study, compound identification was carried out by accurate mass matching combined with MS/MS fragment ion comparison. The specific identification criteria were as follows: the compound was detected in all tested samples; the mass error between the measured and theoretical values of precursor ions was within ±10 ppm; and the mass error between the measured product ions and the reference values reported in the literature or databases was within ±10 ppm. Compounds meeting all three criteria were considered reliably identified.

#### 4.5.2. Quantitative MS Analysis

Quantitative analysis was performed using a Shimadzu 8050 triple quadrupole mass spectrometer coupled with a Shimadzu LC-30 AD UPLC system (Shimadzu, Kyoto, Japan). The mass spectrometer was operated in positive ionization mode with multiple reaction monitoring (MRM). The interface voltage was set to 3.0 kV. Gas flows were as follows: nebulizing gas, 3.0 L/min; heating gas, 10.0 L/min; drying gas, 10.0 L/min. Temperatures were set as follows: desolvation line (DL), 200 °C; interface, 300 °C; and heating block, 400 °C. Optimized MRM conditions for the three cyclodipeptides are summarized in Appendix Table A1. The precursor-to-product ion transitions and their optimized collision energies for the MRM analysis of cyclo(Pro-Leu), cyclo(Phe-Pro), and cyclo(Leu-Phe) were established via direct infusion. The quantitative analysis utilized the following ion transitions: 211.1 → 70.1 for cyclo(Pro-Leu), 245.1 → 120.15 for cyclo(Phe-Pro), and 261.2 → 120.1 for cyclo(Leu-Phe).

Chromatographic separation was carried out on a Waters CORTECS T3-C18 column (150 mm × 4.6 mm, 2.7 µm) (Waters Corporation, Milford, MA, USA). The flow rate was 0.5 mL/min, the column temperature was maintained at 35 °C, and the injection volume was 1 µL. The mobile phase consisted of (A) methanol and (B) water containing 0.1% formic acid. A gradient elution program was applied: 40% to 80% A from 0 to 12 min.

### 4.6. Prediction of Anti-Inflammatory Components in the Ethyl Acetate Fraction

Candidate small molecules were retrieved from the PubChem database and downloaded in SDF format as 2D structures. Their geometries were subsequently optimized using Chem3D and saved in .mol2 format. Using AutoDockTools (version 1.5.7), hydrogen atoms were added to these small molecules and the structures were energetically minimized before being exported in the .pdbqt format. The target protein receptor was selected from the Protein Data Bank (PDB) and downloaded in .pdb format. The native ligand was removed from the receptor structure using PyMOL (version 3.1.0). The prepared receptor was then dehydrated, hydrogenated, and exported in .pdbqt format using AutoDockTools. Molecular docking simulations and binding energy predictions were performed using AutoDock Vina. The final docking poses and interactions were visualized and analyzed using PyMOL.

### 4.7. In Vitro Activity Evaluation

#### 4.7.1. Cell Viability Determination by CCK-8 Assay

RAW 264.7 cells in the logarithmic growth phase were harvested using a cell scraper, gently resuspended, and counted. The cell suspension was diluted with culture medium to a density of 1 × 10^5^ cells/mL and seeded into a 96-well plate at 100 µL per well. After incubation at 37 °C under 5% CO_2_ for 24 h, the culture medium was aspirated. The blank control group received 100 µL of fresh culture medium. Treatment groups were exposed to 100 µL of the EA, n-Bu, or AR fractions at concentrations of 62.5, 125, 250, and 500 mg/mL, or to the PE fraction at concentrations of 15.625, 31.25, 62.5, and 125 mg/mL. The three cyclodipeptides were tested at concentrations of 30, 15, 7.5, and 3.75 µg/mL. To account for solvent effects, control wells containing 2%, 1%, 0.5%, and 0.25% DMSO were included. After 24 h of incubation, the wells were washed twice with PBS. Under light-protected conditions, 100 µL of CCK-8 solution (10% in medium) was added to each well. Following 1.5 h of incubation and 10 min of orbital shaking, the absorbance of each well was measured at 540 nm using a microplate reader. Cell viability was calculated using the formula below. The maximum non-cytotoxic concentration (cell viability > 95%) for each treatment was determined using GraphPad Prism software.

Cell viability = [(As − Ab)*/*(Ac − Ab)] × 100%

As experimental well: contains cell culture medium, CCK-8, and the drug.

Ac control well: contains cell culture medium and CCK-8, without the drug.

Ab blank well: contains only culture medium and CCK-8.

#### 4.7.2. Nitric Oxide (NO) Assay

RAW 264.7 cells were divided into the following groups: blank control, model (LPS), positive control, and *P. ternata* treatment groups. The positive control group was treated with 10 µM DEX [42]. The different polar fraction treatment groups were administered the EA, n-Bu, or AR fractions at concentrations of 12.5, 25, and 50 mg/mL, or the PE fraction at concentrations of 1.25, 2.5, and 5 mg/mL. The cyclodipeptide treatment groups received the three individual cyclodipeptide components at concentrations of 10, 1, and 0.1 ng/mL. After 2 h of incubation, the culture supernatant was aspirated and replaced with fresh medium containing LPS at a final concentration of 1 µg/mL [43]. Following a 24-h incubation, the cell culture supernatant was collected, and the NO concentration was determined using a Griess reagent assay kit (Beyotime Biotechnology, Shanghai, China) according to the manufacturer’s instructions.

### 4.8. Method Validation and Sample Analysis

Based on the ICH Q2(R2) guideline “Validation of Analytical Procedures,” the method was validated for the determination of three cyclic dipeptide components, including specificity, matrix effect, linearity, precision, recovery, stability, and repeatability.

#### 4.8.1. Specificity

The standard solutions of the three reference compounds and the blank solvent were analyzed separately under the conditions specified in Section 4.5.2. No interfering peaks were observed at the retention times corresponding to the analytes in the blank solvent chromatogram compared to those in the standard solution chromatograms.

#### 4.8.2. Matrix Effect

Standard solutions at a series of concentrations (100, 50, 25, 12.5, and 0 ng) were prepared in both the sample matrix solution (the test solution from Section 4.2.3) and pure methanol. These solutions were analyzed according to the conditions in Section 4.5.2. Calibration curves were constructed by plotting the peak area against the concentration for both the matrix-matched standards and the neat solvent standards. The matrix effect (ME) was calculated as the ratio of the slope of the linear regression equation for the matrix-matched standards to that for the neat solvent standards.

#### 4.8.3. Calibration Curve and Limit of Quantification

A series of standard working solutions at different concentrations were prepared from the stock solution and analyzed under the conditions described in Section 4.5.2. Linear calibration curves were established by plotting the peak area of the characteristic fragment ion for each compound against its corresponding concentration (µg/mL). The limit of detection (LOD) was defined as a signal-to-noise ratio (S/N) of 3. The LOQ was described as the lowest concentration in the calibration curve that could be quantitatively measured when the signal-to-noise ratio was >10.

#### 4.8.4. Precision

The instrument repeatability was assessed by injecting the same standard solution six consecutive times under the conditions specified in Section 4.5.2, and the peak areas were recorded.

#### 4.8.5. Recovery

Recovery was evaluated by analyzing nine samples (0.25 g each) of *P. ternata* with known contents of the three cyclodipeptides. The samples were divided into three groups (n = 3 per group), and each group was spiked with a low, medium, or high concentration of a mixed standard solution. The samples were then processed according to the method in Section 4.2.3 and analyzed under the conditions in Section 4.5.2 to calculate the recovery rate.

#### 4.8.6. Stability

The stability of the processed sample was investigated over 24 h. A single batch of sample was prepared and analyzed at 0, 2, 4, 6, 8, 10, 12, and 24 h after preparation under the analytical conditions described in Section 4.5.2.

#### 4.8.7. Repeatability

Repeatability was determined by accurately weighing six portions of the herbal powder (0.5 g each). Six test solutions were prepared in parallel following the procedure in Section 4.2.3 and analyzed according to the conditions in Section 4.5.2.

### 4.9. Data Processing and Statistical Analysis

Data from the in vitro experiments were statistically analyzed using GraphPad Prism software (version 10.6.0). Inter-group comparisons were performed using one-way analysis of variance (ANOVA) followed by Tukey’s honest significant difference (HSD) post hoc test. Data are presented as the mean ± standard error of the mean (S.E.M.), and a *p*-value < 0.05 was considered statistically significant. For the component analysis data, normalization was performed using Mass Profiler Professional (MPP, version 15.1) software with thresholds set at *p* < 0.05 and fold change > 2. Principal component analysis (PCA) was subsequently conducted using SIMCA (version 14.1) software. Data processing and visualization for sample analysis were performed using Origin 2025 software.

## 5. Conclusions

This study systematically compared the chemical composition of different polar fractions derived from *P. ternata* extracts and evaluated their capacity to inhibit NO production. In an LPS-induced RAW 264.7 macrophage inflammation model, the EA fraction exhibited the most potent inhibition of NO generation, indicating its significant anti-inflammatory potential. Consequently, this research focused on identifying characteristic chemical constituents within these polar fractions. This study represents the first systematic evaluation of the anti-inflammatory activity of three cyclodipeptides isolated from *P. ternata*—cyclo(Pro-Leu), cyclo(Phe-Pro), and cyclo(Leu-Phe). Additionally, a simultaneous, accurate, reliable, and highly sensitive quantitative analytical method was established for their simultaneous analysis. By systematically comparing the chemical profiles and bioactivity differences among the polar fractions, this study concludes that the cyclodipeptides present in the EA fraction are key contributors to NO inhibition. These findings provide a novel avenue for elucidating the anti-inflammatory chemical basis of *P. ternata* and establish a scientific foundation for further investigation into its active constituents and mechanisms of action. Furthermore, this work offers theoretical support and practical guidance for the future development and therapeutic utilization of related anti-inflammatory components.

## Figures and Tables

**Table 1:** Chemical constituents identified in different polar fractions of *P. ternata* by UHPLC-Q-TOF-MS/MS.

No.	tR (min)	Identified Result	Molecular Formula	Ion Type	Measured *m*/*z*	Theoretical *m*/*z*	Mass Error (ppm)	*m*/*z* MS/MS	Main Fraction	Reference
1	11.95	*N*-(2-hydroxyethyl)eicosapentaenamide	C_22_H_37_NO_3_	[M + H]^+^	364.2834	364.2846	−3.29	346.274	PE	[27]
2	16.7	1-ethyl-2-hydroxy-4-oxo-*N′*-palmitoyl-1,4-dihydro-3-quinolinecarbohydrazide	C_22_H_35_NO_2_	[M + H]^+^	346.2738	346.2741	−0.87	128.1065, 110.0967	PE	[28]
3	16.79	1-ethyl-2-hydroxy-4-oxo-*N′*-palmitoyl-1,4-dihydro-3-quinolinecarbohydrazide	C_22_H_35_NO_2_	[M + H]^+^	346.2746	346.2741	1.44	128.1064, 110.0961	PE	[27]
4	18.14	semiplenamide	C_23_H_39_NO_2_	[M + H]^+^	362.3050	362.3054	−1.10	344.2964	PE	[27]
5	19.5	2-(1-azepanylmethyl)-1-vinylcyclododecanol	C_21_H_39_NO	[M + H]^+^	322.3100	322.3104	−1.24	322.3103, 304.2987	PE	[28]
6	19.72	(2*S*,3*S*,4*R*)-2-amino-4-dihydroxyoctadecyl β-d-galactoside	C_24_H_49_NO_8_	[M + H]^+^	480.3530	480.3531	−0.21	462.3408, 444.3326, 342.2998, 206.1019	PE	[27]
7	19.81	*N*-(2-hydroxyethyl)octadecanamide	C_20_H_41_NO_2_	[M + H]^+^	328.3205	328.3210	−1.52	310.3096	PE	[27]
8	19.85	2-amino-octadec-4-yne-1,3-diol	C_18_H_35_NO_2_	[M + H]^+^	298.2737	298.2741	−1.34	282.2731, 262.2527	PE	[27]
9	19.96	2-aminooct-4-yne-1,3-diol	C_18_H_35_NO_2_	[M + H]^+^	298.2738	298.2741	−1.01	280.2625, 250.2527	PE	[28]
10	20.39	phytosphingosine	C_18_H_39_NO_3_	[M + H]^+^	318.3003	318.3003	0.00	264.2677, 282.2784, 300.2892	PE	[28]
11	20.51	Mitoridine	C_21_H_35_NO	[M + H]^+^	318.2799	318.2791	2.51	256.2633	PE	[27]
12	21.89	(2*S*)-2-(dodecylamino)-3-phenyl-1-propanol	C_21_H_37_NO	[M + H]^+^	320.2944	320.2948	−1.25	302.2830, 91.0541	PE	[27]
13	22.27	dihydrosphingosine	C_18_H_39_NO_2_	[M + H]^+^	302.3051	302.3054	−0.99	284.2944	PE	[27]
14	23.02	bis(*N*,*N*-diethylethanaminium)(1*S*)-2-acetamido-1,5-anhydro-2-deoxy-1-[(*R*)-hydroxy(phosphonato)methyl]-d-glucitol	C_21_H_48_N_3_O_9_P	[M + H]^+^	518.3236	518.3201	6.75	459.2407, 313.2724, 146.9789, 104.1069	PE	[27]
15	23.02	LPC(18:3)	C_26_H_48_NO_7_P	[M + H]^+^	518.3263	518.3241	4.24	500.3112, 184.0731, 104.1070	PE	[27]
16	24.03	LPC(16:2)	C_24_H_48_NO_7_P	[M + H]^+^	494.3241	494.3241	0.00	476.3119, 184.0731, 104.1071	PE	[27]
17	24.61	3-{[(2-aminoethoxy)(hydroxy)phosphoryl]oxy}-2-hydroxypropyl-9,12-octadecadienoate	C_23_H_44_NO_7_P	[M + H]^+^	478.2931	478.2928	0.63	460.2841, 337.2737, 306.2785	PE	[27]
18	24.78	LPC(18:2)	C_26_H_50_NO_7_P	[M + H]^+^	520.3409	520.3398	2.11	502.3269, 443.2524, 337.2712, 258.1092, 184.0730	PE	[27]
19	24.82	2-hydroxy-5,8,11,14,17-icosapentaenoyloxy propyl 2-(thimethylammonio)ethyl phosphate	C_28_H_48_NO_7_P	[M + H]^+^	542.3217	542.3241	−4.43	483.2482, 337.2726, 146.9811, 104.1069	PE	[27]
20	25.29	monolinolenin	C_21_H_36_O_4_	[M + H]^+^	353.2688	353.2686	0.57	335.2586, 261.2206, 243.2095	PE	[27]
21	25.3	*N*-(2-hydroxyethoxy)ethyl-icosa-5,8,11,14-tetraenamide	C_23_H_43_NO_2_	[M + H]^+^	366.3364	366.3367	−0.82	307.2619	PE	[27]
22	25.67	3-hydroxypropyl palmitateglc-glucosamine	C_31_H_61_O_14_N	[M + H]^+^	672.4165	672.4165	0.00	313.2741, 239.2362, 163.0610	PE	[27]
23	25.73	2,3-dihydroxypropyl(9*Z*,12*Z*,15*Z*)-9,12,15-octadecatrienoate-hexose-hexose	C_33_H_56_O_14_	[M + H]^+^	677.3708	677.3743	−5.17	515.3191, 353.2164	PE	[27]
24	25.99	3-{[(2-aminoethoxy)(hydroxy)phosphoryl]oxy}-2-hydroxypropyl palmitate	C_21_H_44_NO_7_P	[M + H]^+^	454.293	454.2928	0.44	436.2839, 313.2735, 282.2796	PE	[27]
25	26.2	LPC(16:1)	C_24_H_50_NO_7_P	[M + H]^+^	496.3401	496.3398	0.60	478.3286, 313.2742, 258.1098, 184.0740, 124.9991, 104.1074	PE	[27]
26	26.67	monolinolein	C_21_H_38_O_4_	[M + H]^+^	355.2843	355.2843	0.00	337.2708, 263.2306, 245.2255	PE	[27]
27	27.06	3-{[(2-aminoethoxy)(hydroxy)phosphoryl]oxy}-2-hydroxypropyl-9,12-octadecadienoate	C_23_H_46_NO_7_P	[M + H]^+^	480.3085	480.3085	0.00	462.2966, 339.2883, 308.2967	PE	[27]
28	27.1	linolenic acid	C_18_H_30_O_2_	[M + H]^+^	279.2313	279.2319	−2.15	131.0838	PE	[28]
29	27.21	LPC(18:1)	C_26_H_52_NO_7_P	[M + H]^+^	522.3555	522.3554	0.19	504.3440, 184.0731, 124.9989, 104.1067	PE	[27]
30	27.22	propyl 2-(trimethylammonio)ethyl phosphate	C_28_H_50_NO_7_P	[M + H]^+^	544.3374	544.3398	−4.41	485.2640, 339.2853, 104.1069	PE	[27]
31	29.33	palmitic acid	C_16_H_32_O_2_	[M + H]^+^	257.2468	257.2475	−2.72	149.1287	PE	[28]
32	29.35	monopalmitin	C_19_H_38_O_4_	[M + H]^+^	331.2836	331.2843	−2.11	313.2724, 239.2369	PE	[28]
33	29.35	(2*S*)-2,3-dihydroxypropyl hexadecanoate hexoside	C_25_H_48_O_9_	[M + H]^+^	493.334	493.3371	−6.28	239.2358, 331.2831, 493.3340	PE	[28]
34	30.44	LPC(18:0)	C_26_H_54_NO_7_P	[M + H]^+^	524.371	524.3711	−0.19	506.3589, 184.0733, 104.1069	PE	[27]
35	30.89	linoleoylethanolamide	C_20_H_37_NO_2_	[M + H]^+^	324.2892	324.2897	−1.54	306.2765, 245.2266	PE	[27]
36	32.86	crucigasterin E	C_18_H_33_NO	[M + H]^+^	280.2629	280.2635	−2.14	280.2600, 262.2487	PE	[28]
37	33.71	stigmasterol	C_29_H_48_O	[M + H]^+^	413.3755	413.3778	−5.56	395.3643, 413.3755	PE	[28]
38	33.99	linolenic acid	C_18_H_30_O_2_	[M − H]^−^	277.2185	277.2173	4.33	97.9798, 79.9592	PE	[29]
39	36.51	linoleic acid	C_18_H_32_O_2_	[M + H]^+^	281.2466	281.2475	−3.20	245.2254	PE	[28]
40	38.77	palmitic acid	C_16_H_32_O_2_	[M − H]^−^	255.2342	255.2330	4.70	116.9293, 114.0090	PE	[29]
41	38.79	isopalmitic acid	C_16_H_32_O_2_	[M − H]^−^	255.2342	255.2330	4.70	186.0706, 116.9293	PE	[29]
42 *	1.76	succinate	C_4_H_6_O_4_	[M − H]^−^	117.0196	117.0193	2.56	99.0088, 73.0297	EA	[29]
43 *	6.98	cyclo(Pro-Leu)	C_11_H_18_N_2_O_2_	[M + H]^+^	211.1439	211.1441	−0.95	70.0654, 138.1491, 72.0440	EA	-
44 *	8.63	cyclo(Phe-Pro)	C_14_H_16_N_2_O_2_	[M + H]^+^	245.1286	245.1285	0.41	120.0803, 70.0654, 154.0740	EA	-
45	9.08	BruceineA	C_26_H_34_O_11_	[M − H]^−^	521.2066	521.2028	7.29	101.0248, 89.0248, 71.0140, 59.0139	EA	[29]
46	10.04	nonanedioic acid	C_9_H_16_O_4_	[M − H]^−^	187.0986	187.0976	5.34	187.8574, 125.0970, 97.0658, 137.0247	EA	[30]
47	10.78	protocatechualdehyde	C_7_H_6_O_3_	[M − H]^−^	137.0247	137.0244	2.19	137.0247	EA	[30]
48	10.83	Leu-Tyr	C_15_H_22_N_2_O_4_	[M − Na]^−^	271.168	271.1676	1.48	271.1601, 171.0489, 140.0717	EA	[30]
49 *	11.75	cyclo (Leu-Phe)	C_15_H_20_N_2_O_2_	[M + H]+	261.1593	261.1598	−1.91	120.0803, 103.0538, 86.0964	EA	-
50	14.65	10-Curcumin	C_21_H_34_O_4_	[M + H]^+^	351.2524	351.2530	−1.71	95.0858, 81.0697, 67.0538	EA	[29]
51	32.75	unknown	unknown	[M + H]^+^	448.2903	448.2903	0.00	185.0803, 157.0140, 129.0174	EA	-
52 *	1.51	uridine	C_9_H_12_N_2_O_6_	[M − H]^−^	243.064	243.0623	6.99	200.9968, 152.0394, 110.0253, 82.0282	n-Bu	[29]
53 *	1.54	adenosine	C_10_H_13_N_5_O_4_	[M + H]^+^	268.1048	268.1040	2.98	137.0643, 136.0615, 110.0694	n-Bu	[29]
54 *	1.64	guanine	C_5_H_5_N_5_O	[M + H]^+^	152.0565	152.0567	−1.32	135.0298, 134.0466, 110.0347	n-Bu	[29]
55 *	1.65	guanosine	C_10_H_13_N_5_O_5_	[M + H]^+^	284.099	284.0989	0.35	152.0564, 135.0298, 110.0350	n-Bu	[29]
56	3.14	ellipticine	C_17_H_14_N_2_	[M + H]^+^	247.1254	247.1230	9.71	72.0799, 70.0646	n-Bu	[29]
57	3.76	l-tryptophan	C_11_H_12_N_2_O_2_	[M + H]^+^	205.0972	205.0972	0.00	188.0704, 146.0598, 144.0801, 118.0648	n-Bu	[29]
58 *	3.76	tryptophan	C_11_H_12_N_2_O_2_	[M + H]^+^	205.0972	205.0972	0.00	188.0704, 146.0598	n-Bu	[29]
59	3.77	3-amino-2-naphthoic acid	C_11_H_9_NO_2_	[M + H]^+^	188.0706	188.0706	0.00	170.0591	n-Bu	[27]
60	5.22	norharman	C_11_H_8_N_2_	[M + H]^+^	169.0761	169.0760	0.59	95.0847, 70.0643	n-Bu	[29]
61	6.64	apigenin 6-C-glucosyl-8-C-arabinoside	C_26_H_28_O_14_	[M + H]^+^	565.1537	565.1552	−2.65	547.1450, 529.1329, 511.1230	n-Bu	[27]
62	7.54	tridecanoylglycine	C_15_H_29_NO_3_	[M + H]^+^	272.2225	272.2220	1.84	254.2112	n-Bu	[27]
63	9.37	pikromycin	C_28_H_47_NO_8_	[M + H]^+^	526.3368	526.3374	−1.14	508.3252, 364.2819	n-Bu	[27]
64	1.06	l-arginine	C_6_H_14_N_4_O_2_	[M − H]^−^	173.1049	173.1044	2.89	156.0781, 131.0824, 114.0563	AR	[29]
65	1.11	glucose	C_6_H_12_O_6_	[M − H]^−^	179.0569	179.0561	4.47	179.1120, 85.0275, 71.0141	AR	[30]
66	1.12	l-serine	C_3_H_7_NO_3_	[M − H]^−^	104.0354	104.0353	0.96	74.0250, 59.0144	AR	[29]
67	1.14	kojibiose	C_12_H_22_O_11_	[M − H]^−^	341.1111	341.1089	6.45	341.1069, 179.0564, 149.0453, 119.0351	AR	[30]
68	1.15	1,4-anhydro-1-[5-carbamoyl-1-(4-nitrophenyl)-1H-pyrazol-3-yl]pentitol	C_15_H_16_N_4_O_7_	[M + H]^+^	365.1058	365.1092	−9.31	203.0522, 185.0418	AR	[27]
69	1.18	Ala-Asp	C_7_H_12_N_2_O_5_	[M + H]^+^	205.0818	205.0819	−0.49	205.0838, 187.0710, 157.0571, 145.0499	AR	[29]
70	1.21	*N*-(4-methyl-2-pentanoic acid)-glutamic acid	C_11_H_19_NO_6_	[M + H]^+^	262.1286	262.1285	0.38	244.1167, 234.1365, 216.1226, 198.1112	AR	[29]
71 *	1.25	methionine	C_5_H_11_NO_2_S	[M + H]^+^	150.0584	150.0583	0.67	133.0499, 104.0533, 56.0501, 61.0111	AR	[29]
72	1.35	malic acid	C_4_H_6_O_5_	[M − H]^−^	133.0149	133.0142	5.26	133.0143, 115.0036, 71.0139, 59.0141	AR	[30]
73	1.49	(2*R*,3*R*)-5-ethoxy-2-[(ethoxyacetyl)amino]-3-methyl-5-oxopentanoic acid	C_12_H_21_NO_6_	[M + H]^+^	276.1445	276.1442	1.09	258.1334, 248.1509	AR	[27]
74 *	1.52	tyrosine	C_9_H_11_NO_3_	[M + H]^+^	182.081	182.0812	−1.10	166.0963, 86.0580, 72.0821	AR	[29]
75	2.39	aconitic acid	C_6_H_6_O_6_	[M − H]^−^	173.0102	173.0092	5.78	129.8062, 111.0084, 85.0277, 67.0187	AR	[29]
76	2.45	phenylalanine	C_9_H_11_NO_2_	[M + H]^+^	166.0864	166.0863	0.60	166.0841, 120.0805, 103.0539, 91.0537	AR	[29]
77	2.59	gentiatibetine	C_9_H_11_NO_2_	[M + H]^+^	166.086	166.0863	−1.81	120.0803, 69.0321	AR	[29]
78	2.88	corynanthine	C_21_H_26_N_2_O_3_	[M + H]^+^	377.1818	377.1836	−4.77	216.1230, 147.0469, 72.0811	AR	[29]
79	4.47	*N*-dodecanoyl-l-serine	C_15_H_29_NO_4_	[M + H]^+^	288.2161	288.2169	−2.78	270.2072	AR	[27]

Note: Compounds marked with an asterisk (*) were verified using reference standards. For compounds without standards, structures were tentatively identified by matching MS/MS data with literature reports. Although these configurations represent the best fit for the data, the existence of alternative stereoisomers cannot be excluded.

**Table 2:** Linear regression data, LOD, and LOQ for the analytes.

Compound	Calibration Equation	Linear Range (ng)	R^2^	LOD (ng/mL)	LOQ (ng/mL)
cyclo(Pro-Leu)	y = 3,732,703x + 8256	1.53–122.63	0.9991	0.21	1.53
cyclo(Phe-Pro)	y = 2,366,934x − 417	1.28–102.38	0.9990	0.20	1.28
cyclo(Leu-Phe)	y = 8,341,267x + 14,044	1.90–152.38	0.9995	0.20	1.90

**Table 3:** Matrix effect for the three cyclodipeptides.

cyclo(Pro-Leu)	Matrix Solution	y = 137,951x + 249,724, R^2^ = 0.9932
	Methanol Solution	y = 132,197x + 405,044, R^2^ = 0.9980
	Matrix Effect (%)	95.83
cyclo(Phe-Pro)	Matrix Solution	y = 45,294x + 42,216, R^2^ = 0.9814
	Methanol Solution	y = 40,388x + 24,805, R^2^ = 0.9870
	Matrix Effect (%)	89.29
cyclo(Leu-Phe)	Matrix Solution	y = 273,664x + 629,037, R^2^ = 0.9964
	Methanol Solution	y = 266,406x + 814,477, R^2^ = 0.9932
	Matrix Effect (%)	97.35

**Table 4:** Precision data for the three cyclodipeptides.

No.	Peak Area of cyclo(Pro-Leu)	Peak Area of cyclo(Phe-Pro)	Peak Area of cyclo(Leu-Phe)
1	85,932	66,577	191,670
2	85,303	66,568	190,289
3	84,909	66,666	191,071
4	85,470	66,275	190,261
5	85,366	66,886	188,016
6	85,605	66,297	189,233
RSD%	0.40	0.35	0.69

**Table 5:** Solution stability of the three cyclodipeptides (0–24 h).

Time (h)	Concentration of cyclo(Pro-Leu)	Concentration of cyclo(Phe-Pro)	Concentration of cyclo(Leu-Phe)
	μg/g	μg/g	μg/g
0	0.8274	1.1126	0.8367
2	0.8084	1.1193	0.8392
4	0.8092	1.1173	0.8361
6	0.8088	1.1224	0.8374
8	0.8079	1.1251	0.8342
10	0.8115	1.1268	0.8363
12	0.8079	1.1370	0.8412
24	0.8114	1.1118	0.8438
AVERAGE	0.8116	1.1215	0.8381
RSD%	0.81	0.74	0.37

**Table 6:** Repeatability data for the three cyclodipeptides.

No.	Concentration of cyclo(Pro-Leu)	Concentration of cyclo(Phe-Pro)	Concentration of cyclo(Leu-Phe)
	μg/g	μg/g	μg/g
1	0.8106	1.1572	0.8441
2	0.8277	1.1226	0.8382
3	0.8184	1.1055	0.8408
4	0.8066	1.1349	0.8444
6	0.8214	1.1397	0.8467
6	0.8178	1.1328	0.8420
AVERAGE	0.8171	1.1321	0.8427
RSD%	0.93	1.53	0.36

**Table 7:** Recovery of the three cyclodipeptides.

Compound	Original Concentration μg	Spiked Concentration μg	Found Concentration μg	Recovery %	RSD %
cyclo(Pro-Leu)	0.207	0.16	0.3647	98.49	2.53
		0.20	0.3949	93.89	1.89
		0.24	0.4353	95.08	1.49
cyclo(Phe-Pro)	0.287	0.224	0.5320	109.37	2.51
		0.28	0.5760	103.23	1.43
		0.336	0.6310	102.37	0.32
cyclo(Leu-Phe)	0.214	0.144	0.3353	84.49	0.72
		0.18	0.3668	85.10	1.03
		0.216	0.4009	86.71	1.54

**Table 8:** Sample Information.

Sample	Origin	Source	Sample	Origin	Source
S1	Gansu province	cultivated	S13	Hebei province	cultivated
S2	Gansu province	cultivated	S14	Hebei province	cultivated
S3	Gansu province	cultivated	S15	Hebei province	cultivated
S4	Gansu province	cultivated	S16	Hebei province	cultivated
S5	Gansu province	cultivated	S17	Hebei province	cultivated
S6	Gansu province	cultivated	S18	Hebei province	cultivated
S7	Gansu province	cultivated	S19	Hebei province	cultivated
S8	Gansu province	cultivated	S20	Hebei province	cultivated
S9	Gansu province	cultivated	S21	Hubei province	wild
S10	Gansu province	cultivated	S22	Hubei province	wild
S11	Hebei province	cultivated	S23	Hubei province	wild
S12	Hebei province	cultivated	S24	Hubei province	wild

## Data Availability

The original contributions presented in this study are included in the article/Supplementary Material. Further inquiries can be directed to the corresponding author.

## Supplementary Materials

The following supporting information can be downloaded at: https://www.mdpi.com/article/10.3390/molecules31081322/s1, MS/MS Spectra of All 79 Identified Compounds and Possible Fragmentation Pathways for Some Compound.

## Appendix A

**Table A1:** Optimized MRM Conditions for Three Cyclodipeptides.

Compound	Precursor Ion (*m*/*z*)	Quantifier (*m*/*z*)	Qualifier (*m*/*z*)	Dwell Time (ms)	Q1 Pre-Bias (V)	Collision Energy (V)	Q3 Pre-Bias (V)	RT/min
cyclo(Pro-Leu)	211.10	70.10	138.15, 72.05	100	−30	−25	−30	6.32
cyclo(Phe-Pro)	245.10	120.15	70.10, 154.10	100	−30	−20	−30	6.93
cyclo(Leu-Phe)	261.20	120.10	103.10, 86.10	100	−20	−20	−26	10.43
