# Supplementary material for: From Activity Screening to Quality Control: UHPLC-MS/MS Analysis of Anti-Inflammatory Cyclodipeptides in Pinellia ternata
Source: Molecules. 2026 Apr 17;31(8):1322. doi: 10.3390/molecules31081322 (PMC13118893; doi:10.3390/molecules31081322)

Supplementary Data: MS/MS Spectra of All 79 Identified Compounds and Possible Fragmentation Pathways for Some Compounds

This supplementary material provides the MS/MS spectra of all compounds listed in Table 1 in the main text, along with the proposed fragmentation pathways for selected compounds, serving as a supplementary illustration of the fragment ion matching process during compound identification.(The fragments within the pink box correspond to the ion fragments listed in Table 1.)

Compound identification was performed using accurate mass matching combined with MS/MS fragment ion comparison. The identification workflow was as follows: accurate precursor ion masses were acquired using UHPLC-Q-TOF, with mass errors within ±10 ppm; meanwhile, the measured fragment ions were compared with reference values from the literature or databases, also within a mass error of ±10 ppm. In addition, candidate compounds were required to be consistently detected across all representative samples to ensure the reliability of the identification results.

Based on the above criteria, compounds meeting all three conditions (mass accuracy, MS/MS fragment ion matching, and consistent detection across samples) were considered reliably identified. In this supplementary material, for some compounds, possible fragmentation pathways are further provided alongside their MS/MS spectra, aiming to offer a more intuitive illustration of fragment ion assignment as a complement to the identification method.

Compound 1

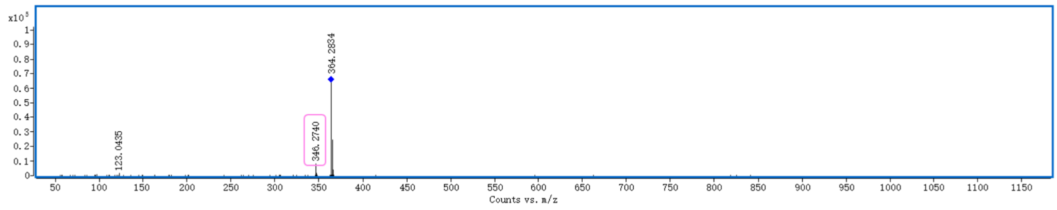

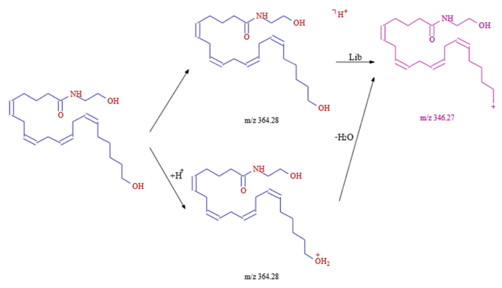

Compound 2

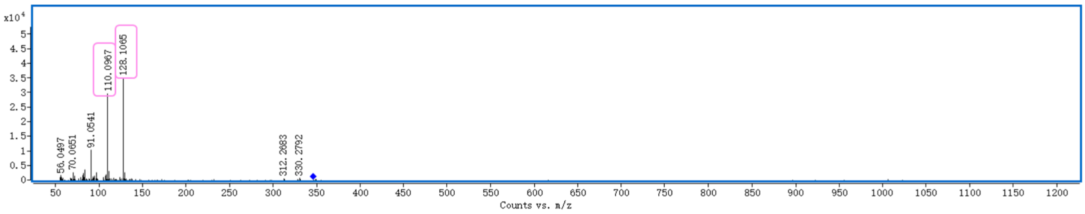

Compound 3

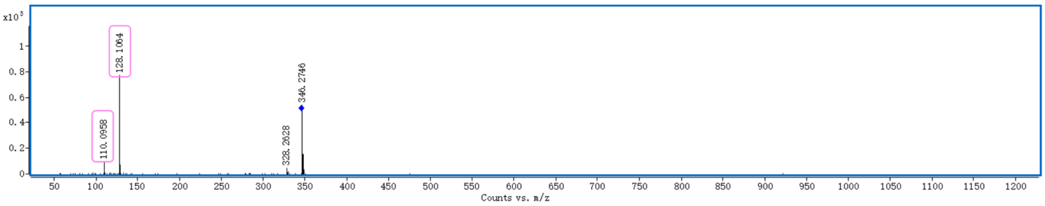

Compound 4

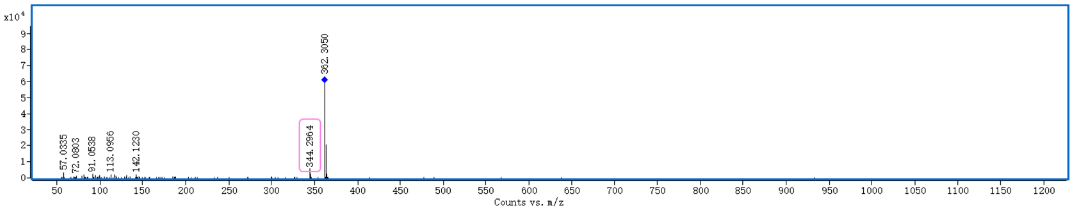

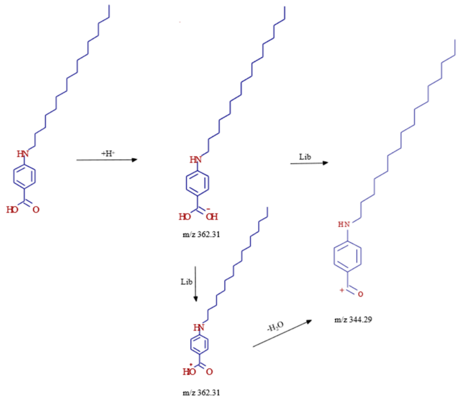

Compound 5

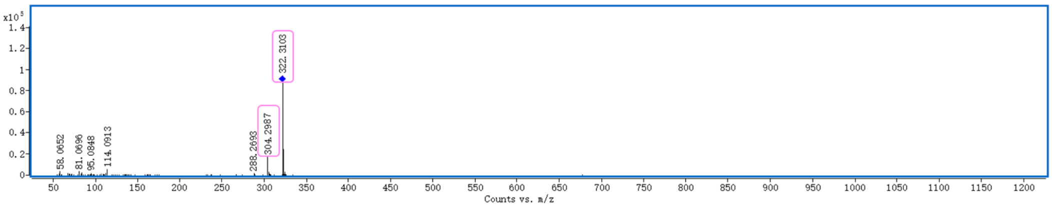

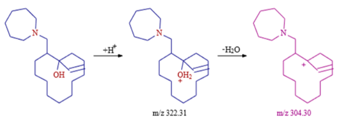

Compound 6

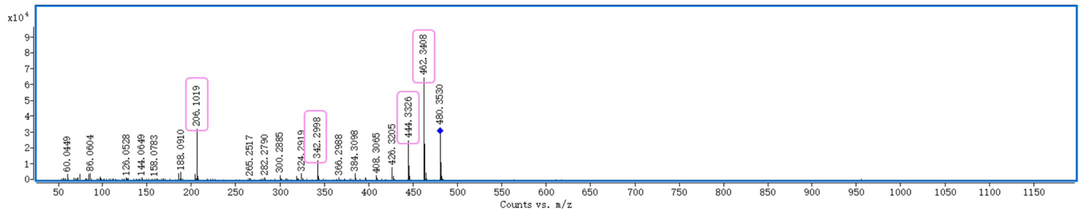

Compound 7

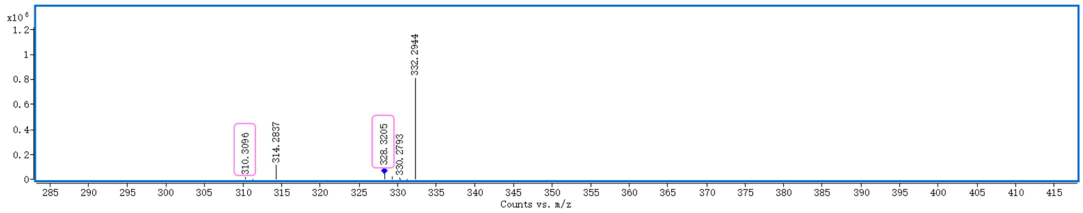

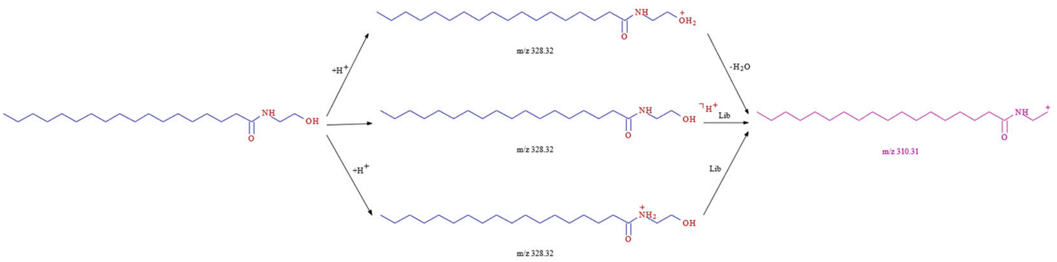

Compound 8

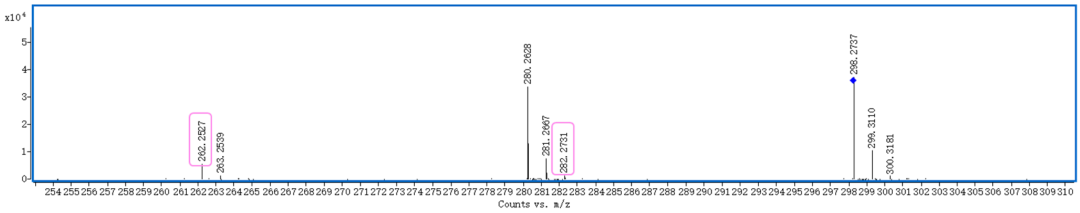

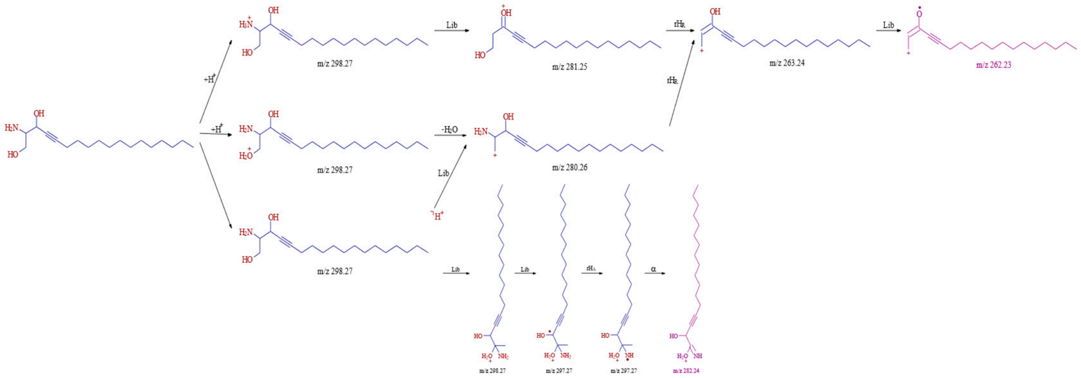

Compound 9

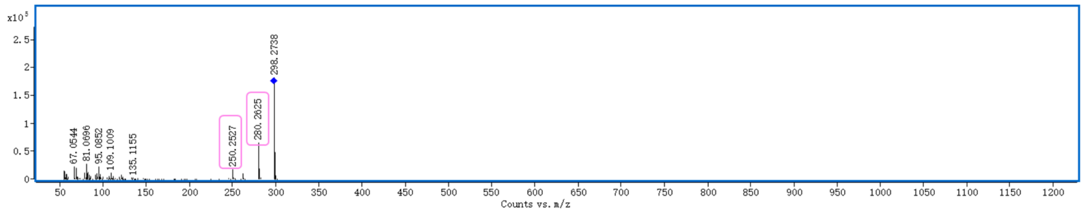

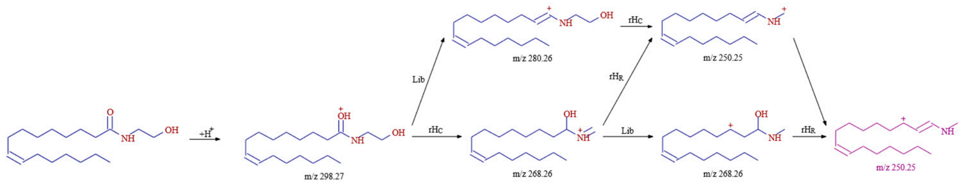

Compound 10

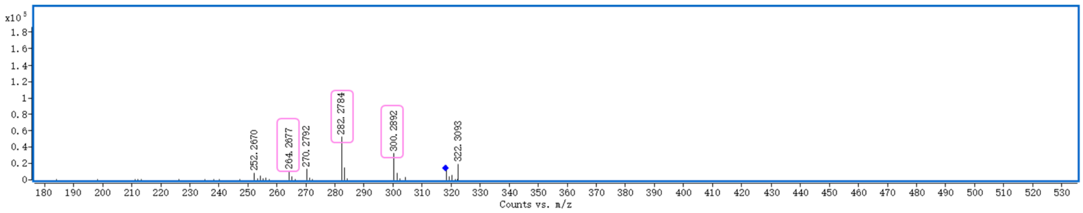

Compound 11

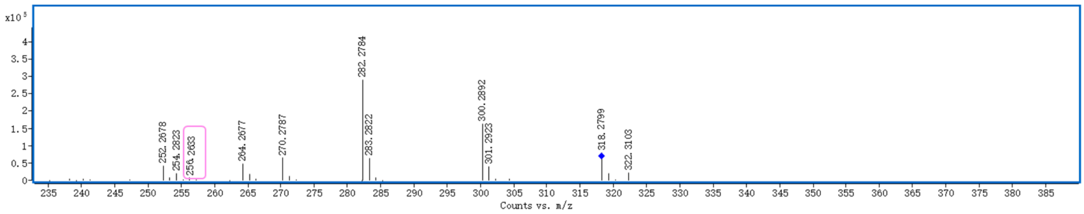

Compound 12

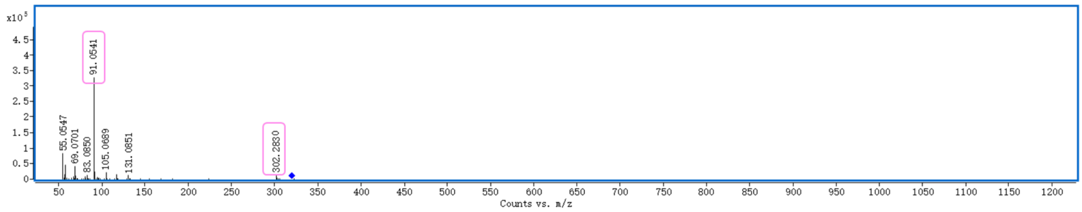

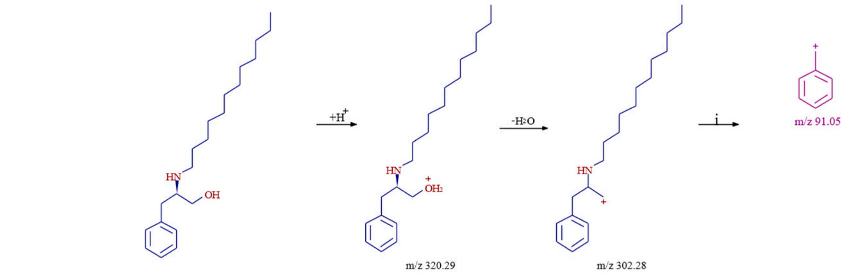

Compound 13

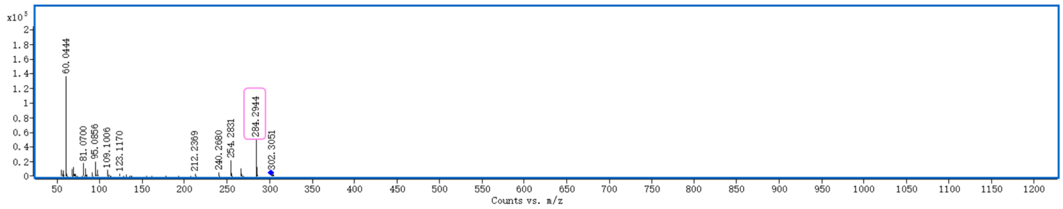

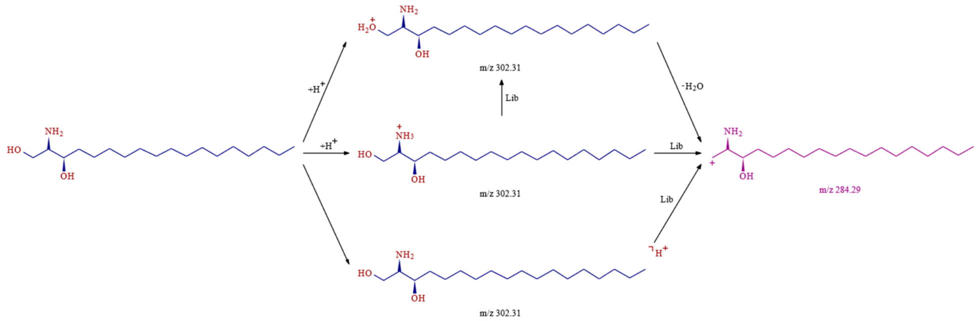

Compound 14

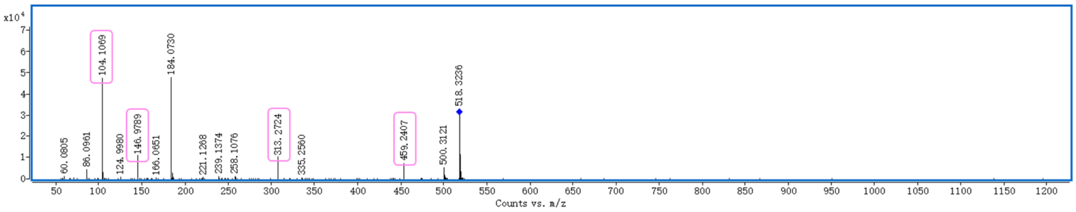

Compound 15

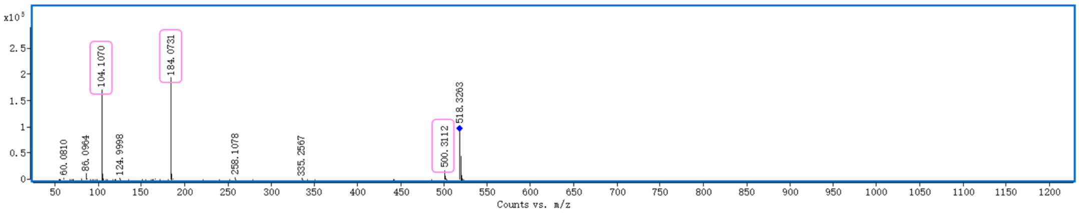

Compound 16

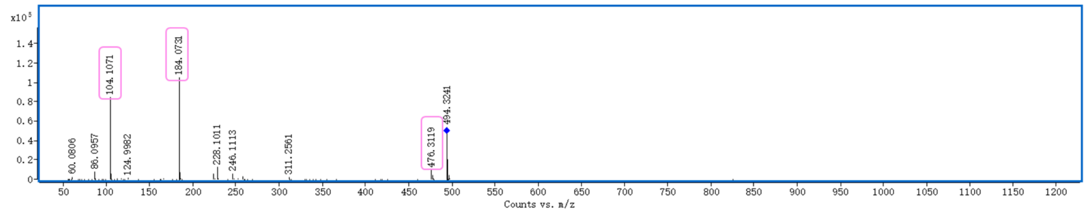

Compound 17

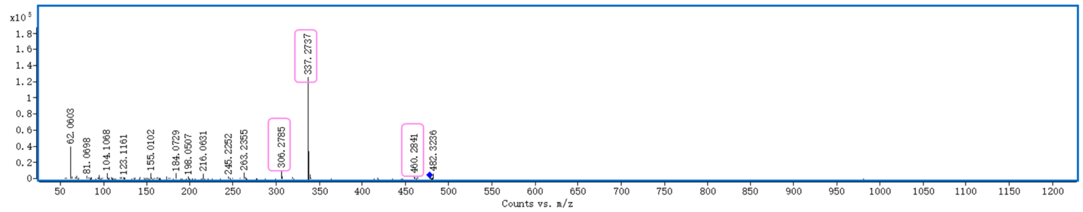

Compound 18

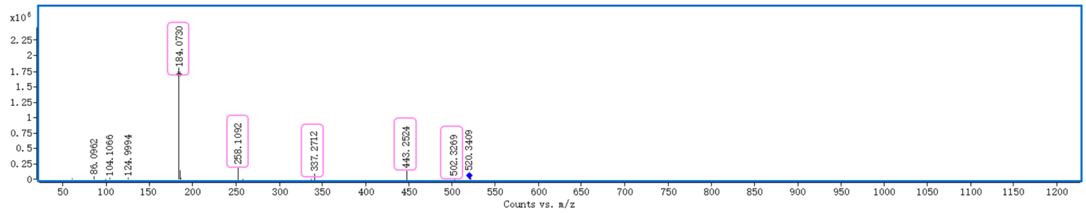

Compound 19

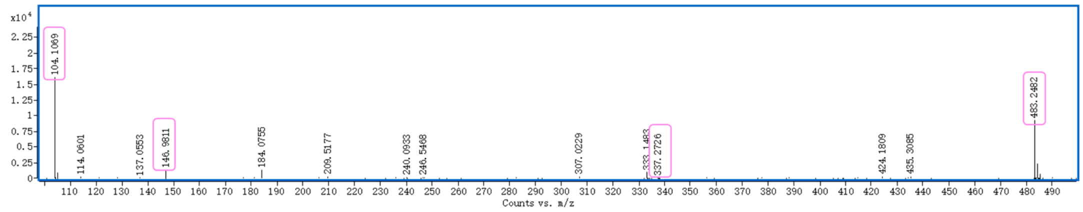

Compound 20

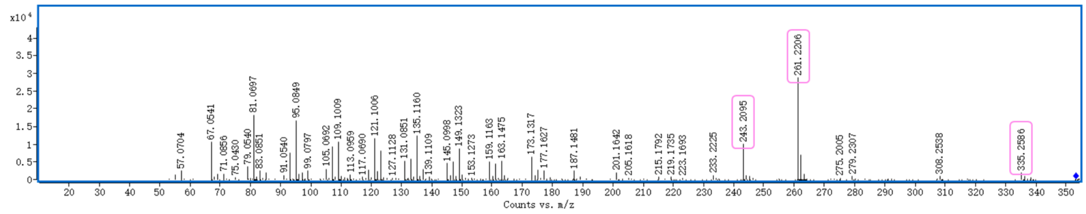

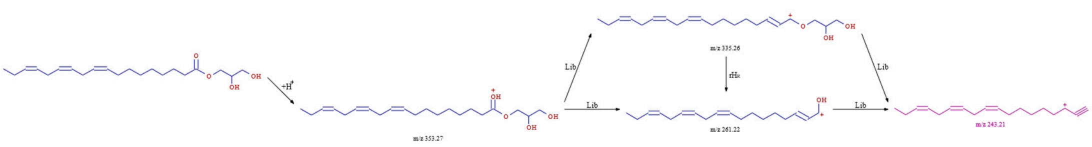

Compound 21

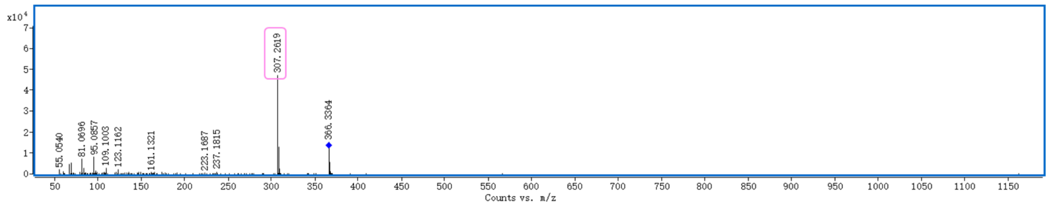

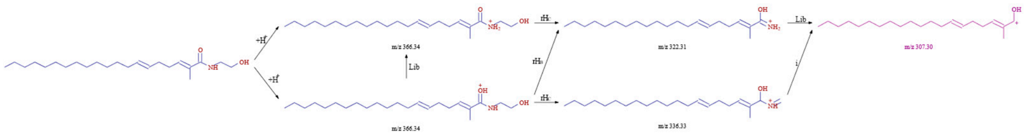

Compound 22

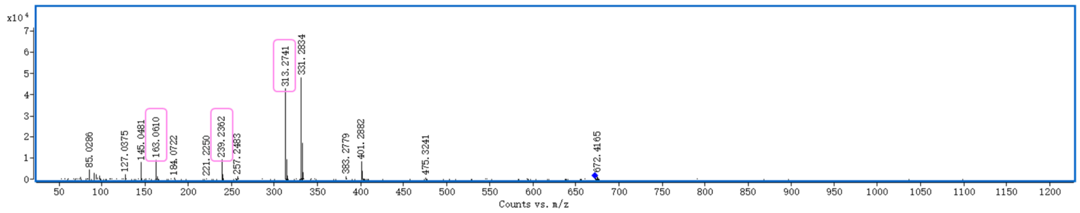

Compound 23

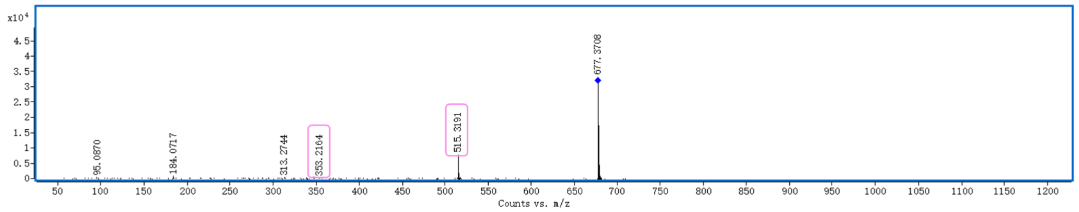

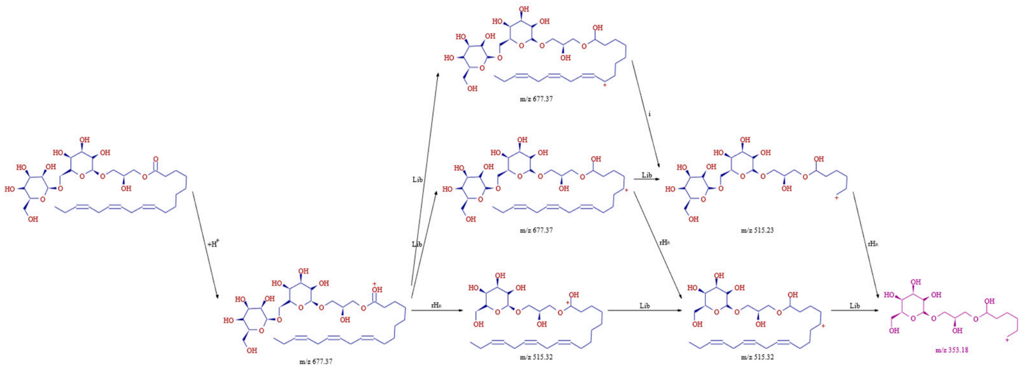

Compound 24

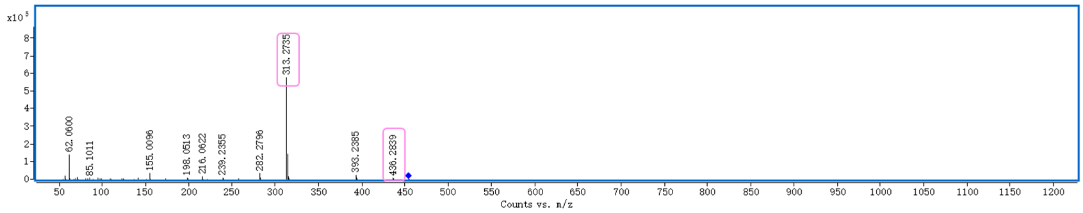

Compound 25

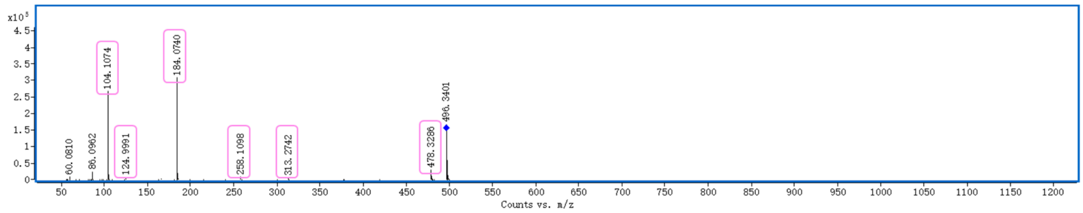

Compound 26

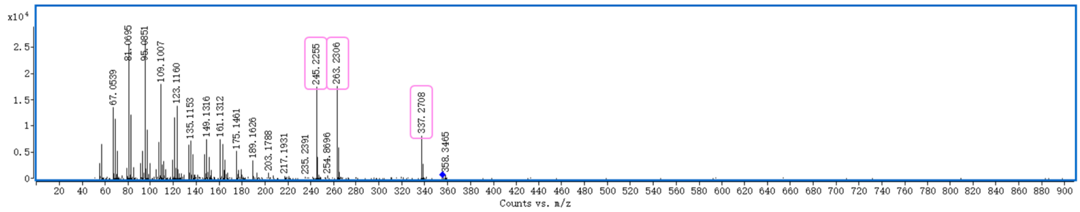

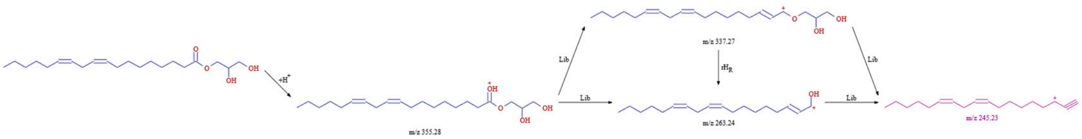

Compound 27

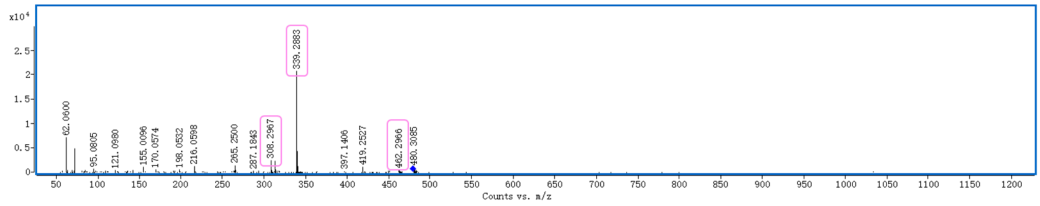

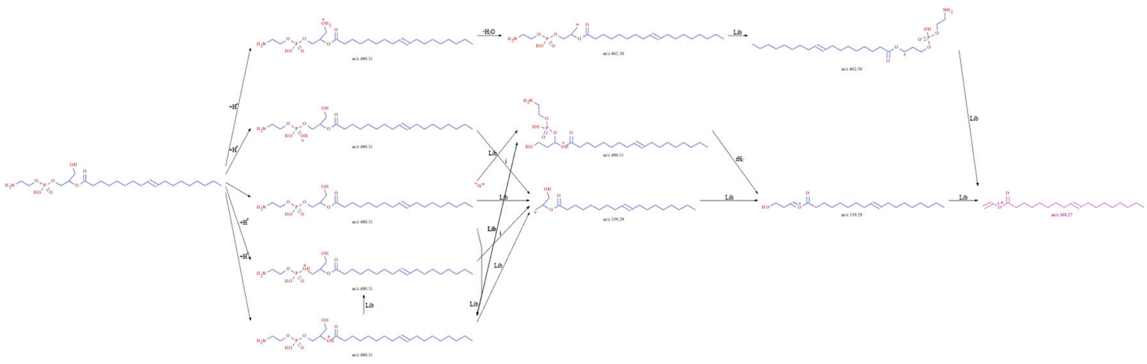

Compound 28

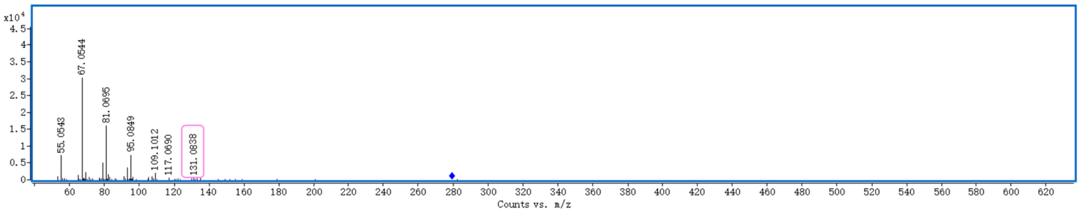

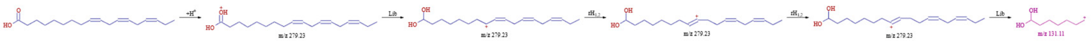

Compound 29

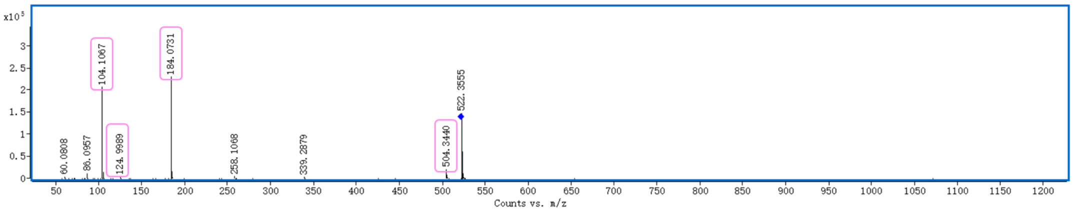

Compound 30

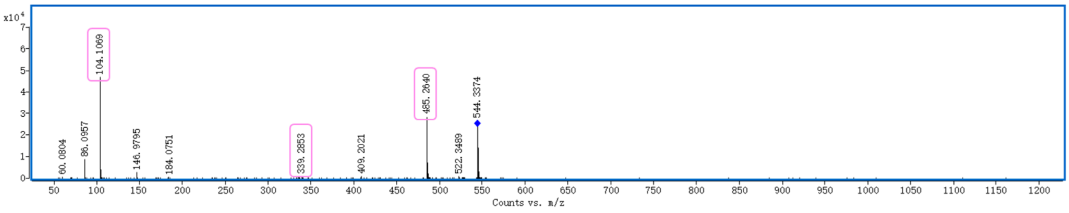

Compound 31

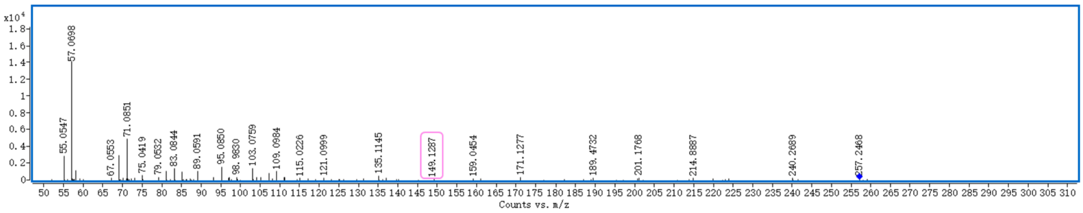

Compound 32

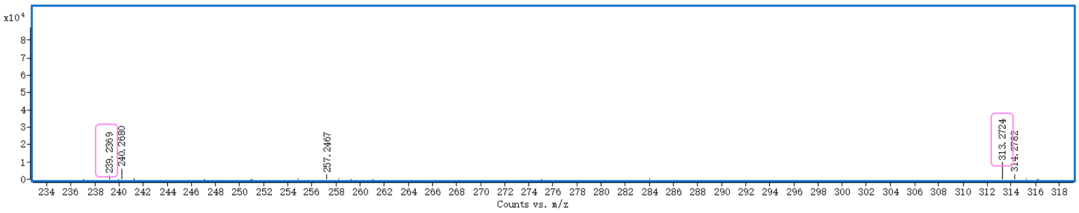

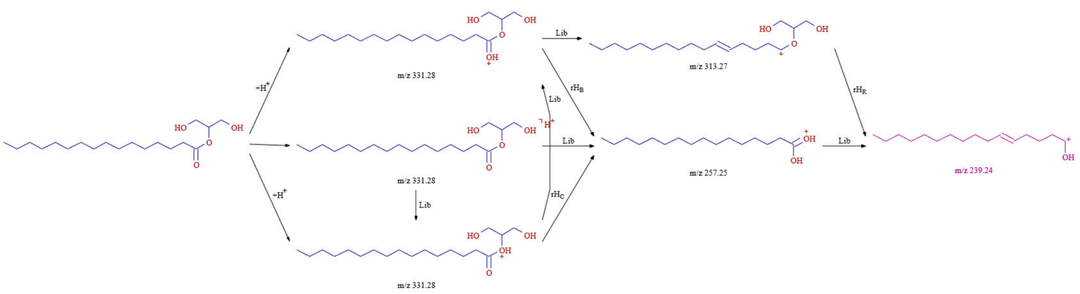

Compound 33

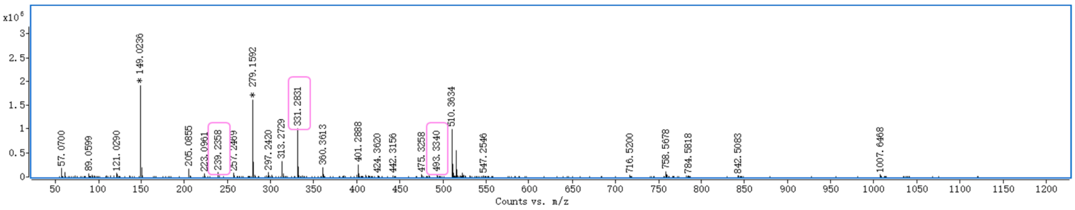

Compound 34

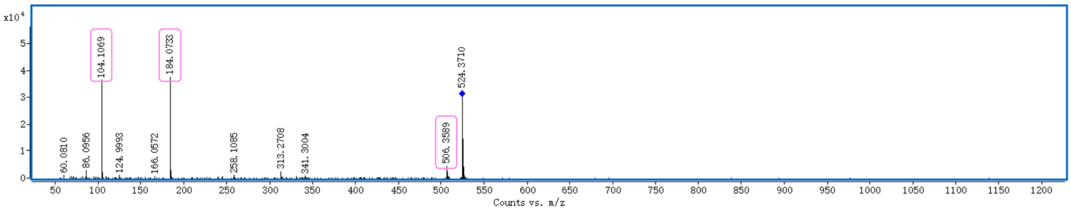

Compound 35

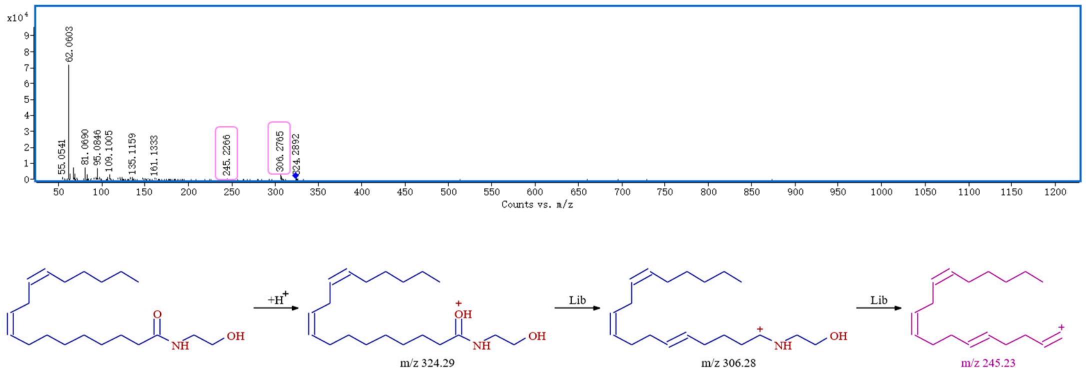

Compound 36

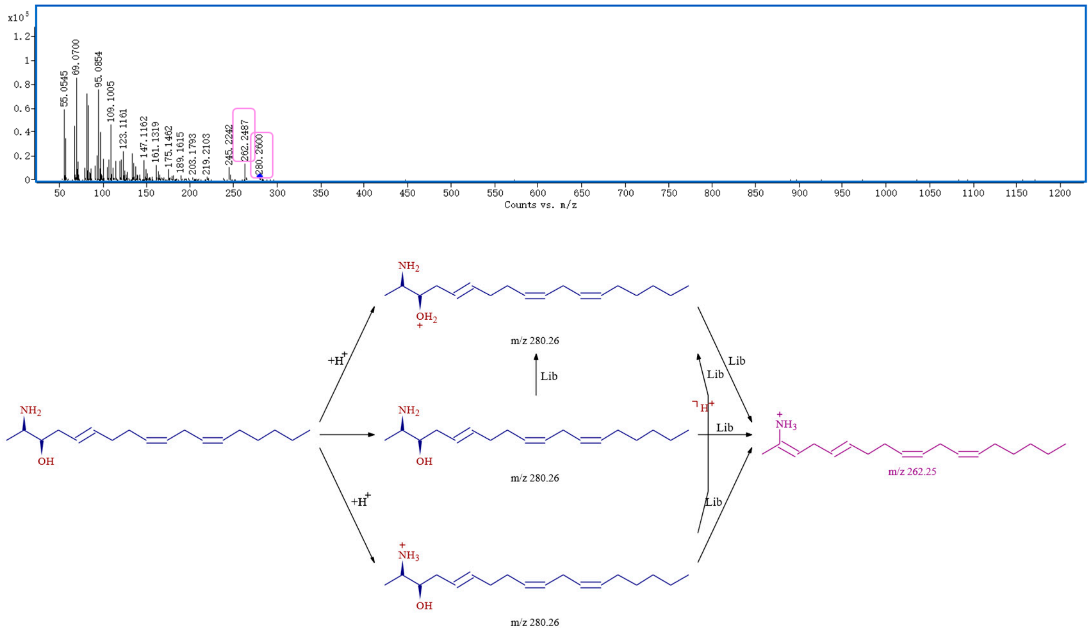

Compound 37

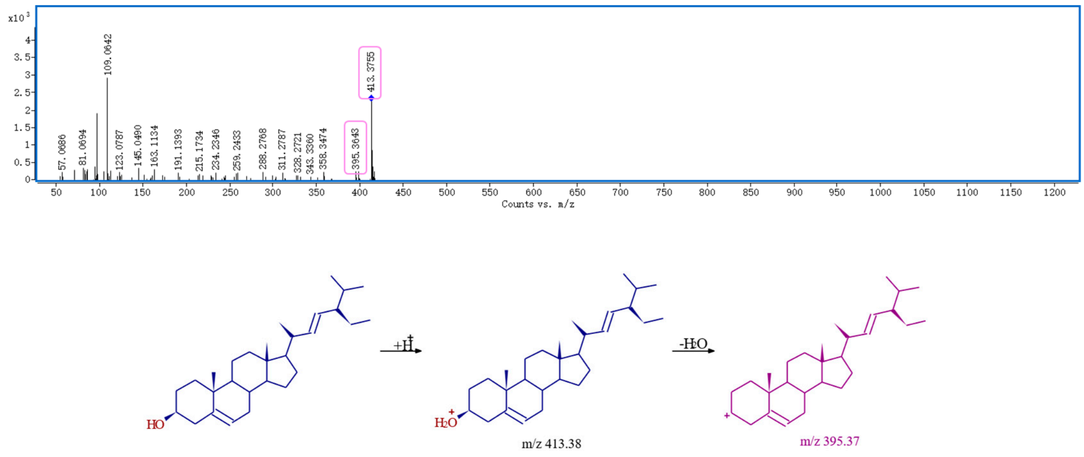

Compound 38

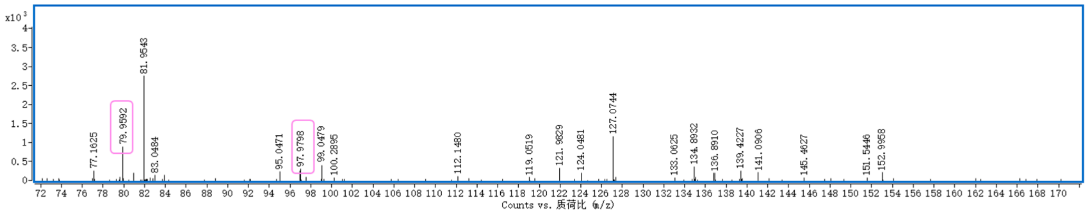

Compound 39

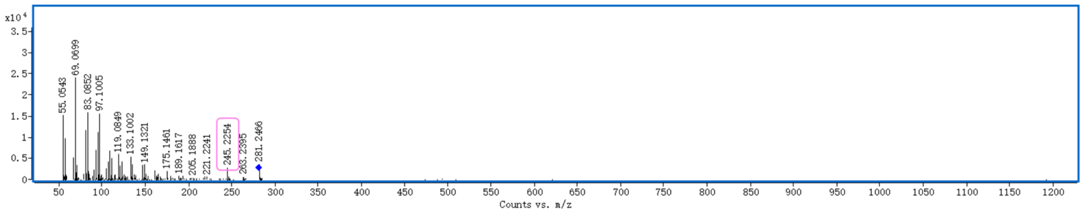

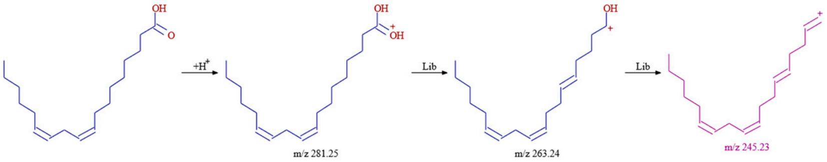

Compound 40

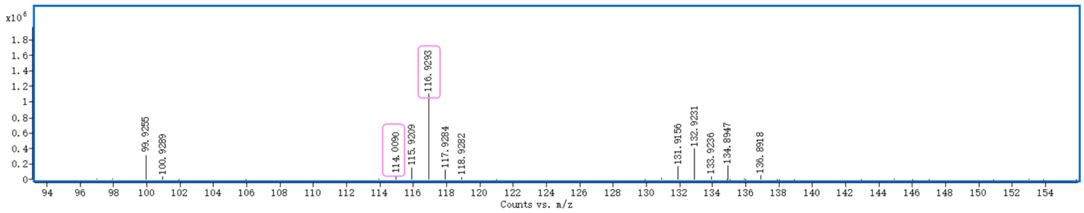

Compound 41

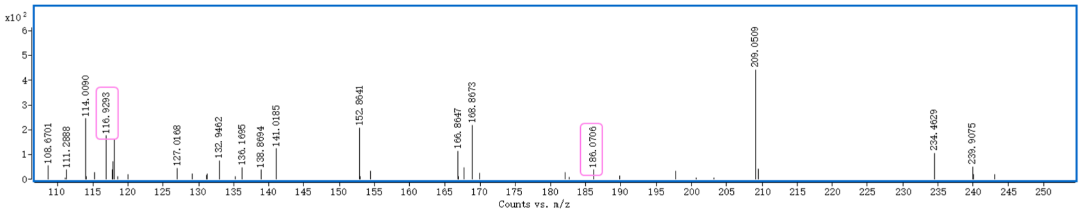

Compound 42

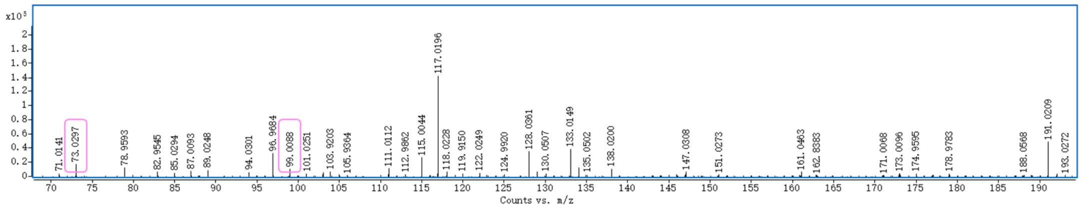

Compound 43

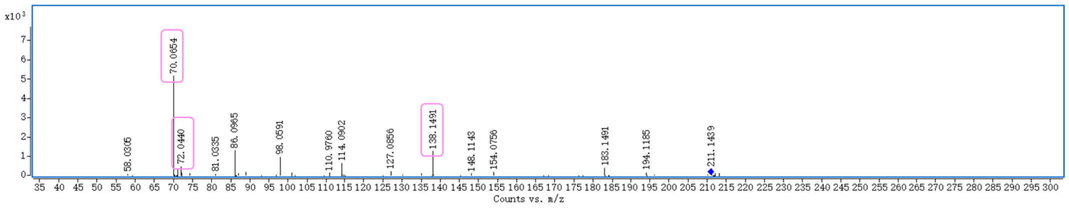

Compound 44

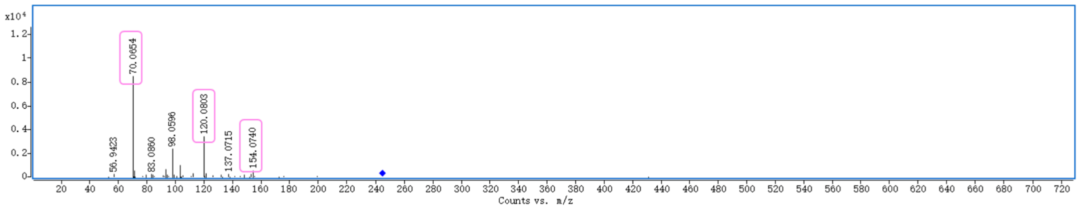

Compound 45

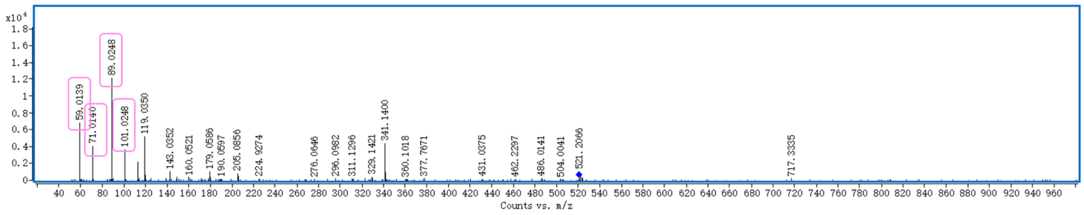

Compound 46

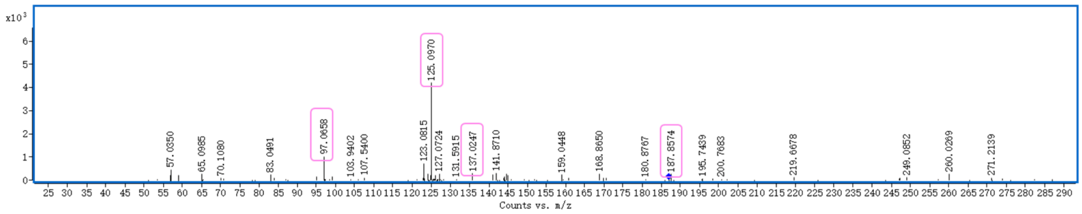

Compound 47

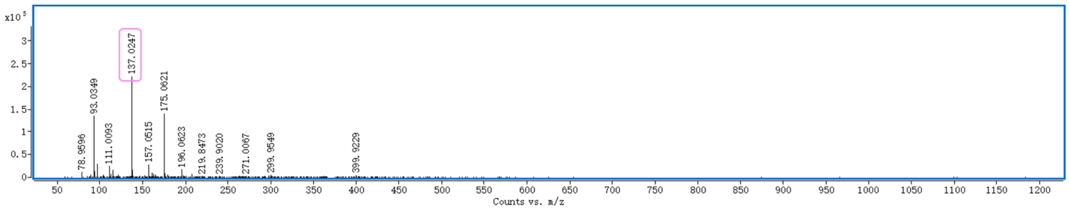

Compound 48

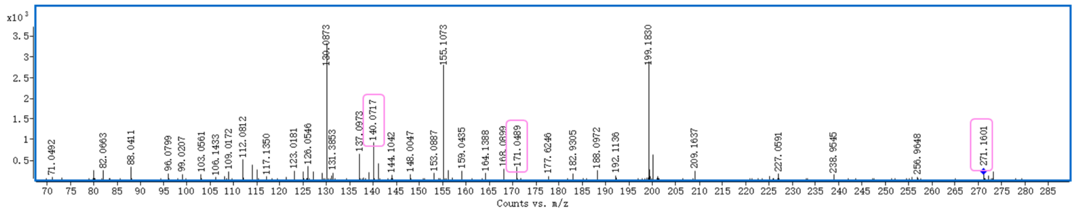

Compound 49

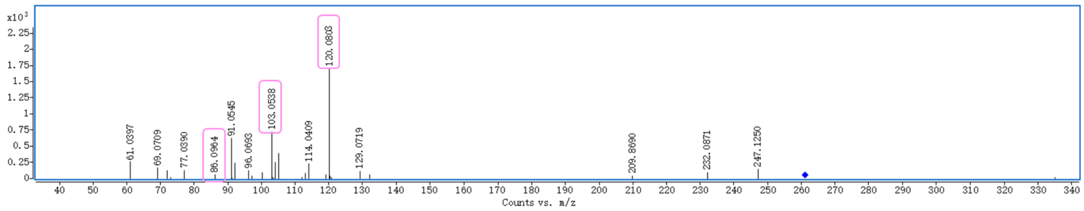

Compound 50

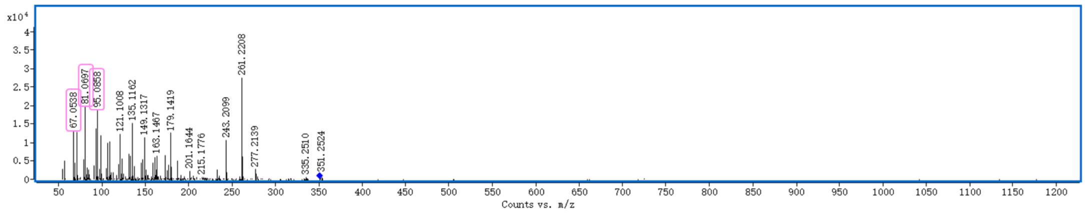

Compound 51

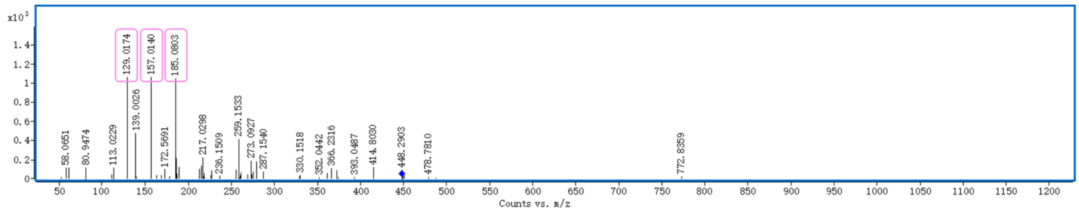

Compound 52

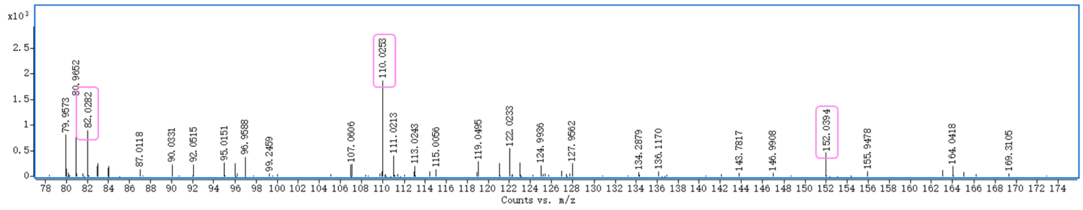

Compound 53

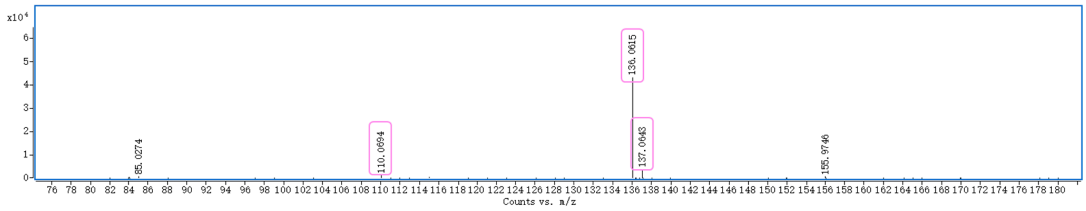

Compound 54

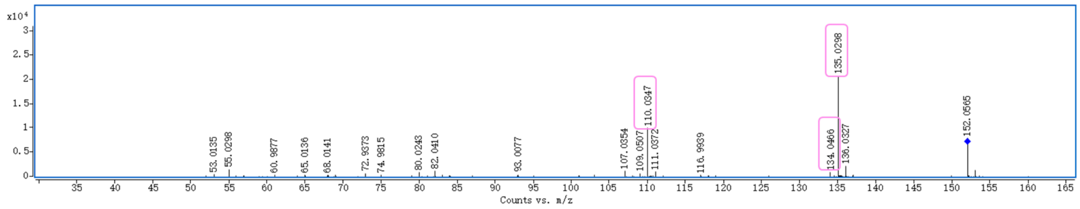

Compound 55

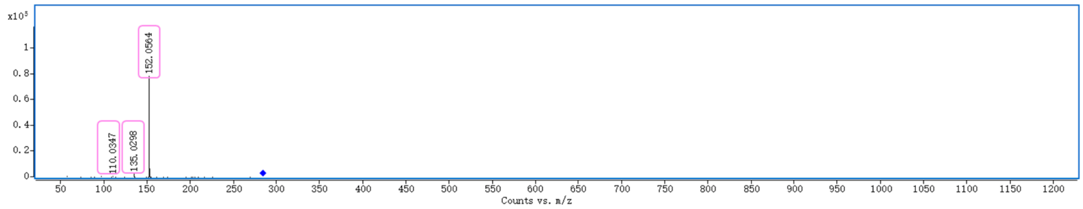

Compound 56

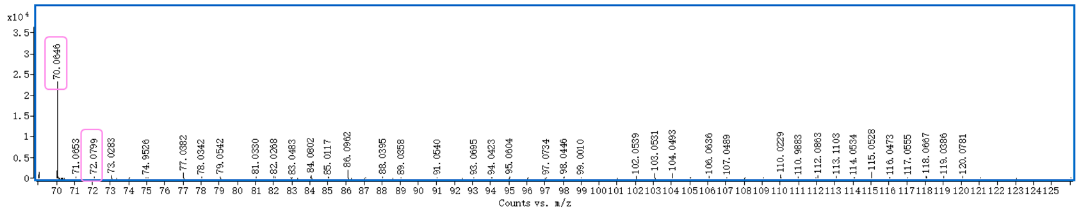

Compound 57

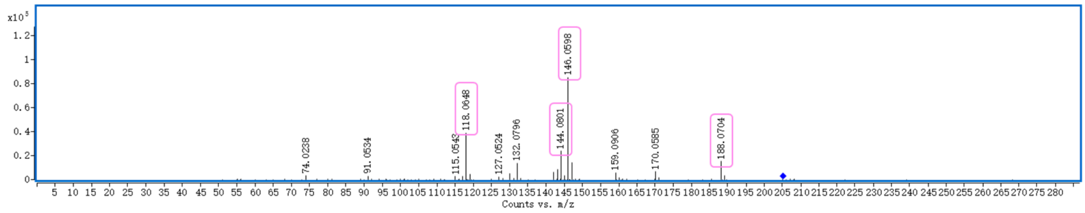

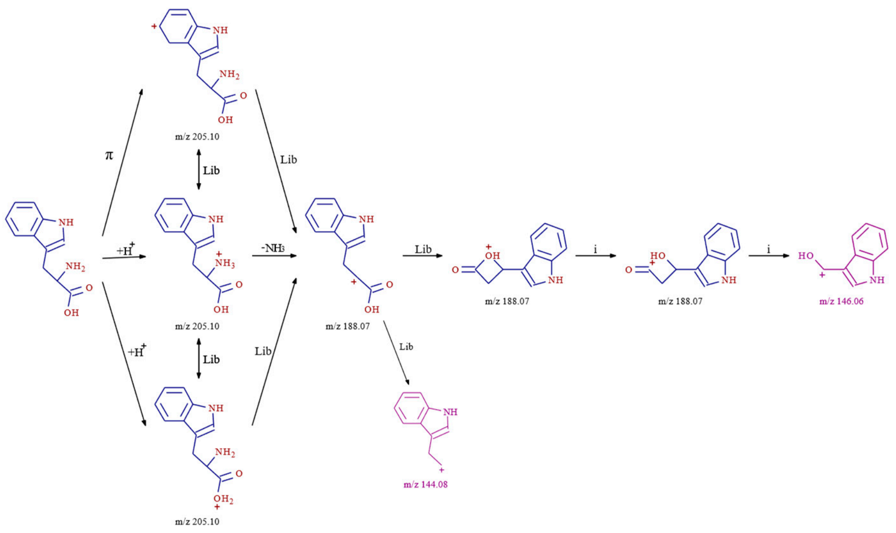

Compound 58

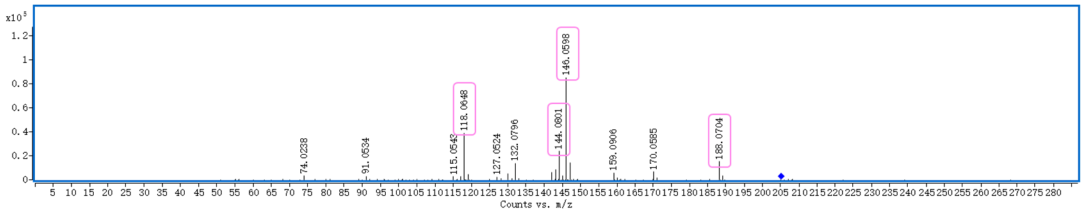

Compound 59

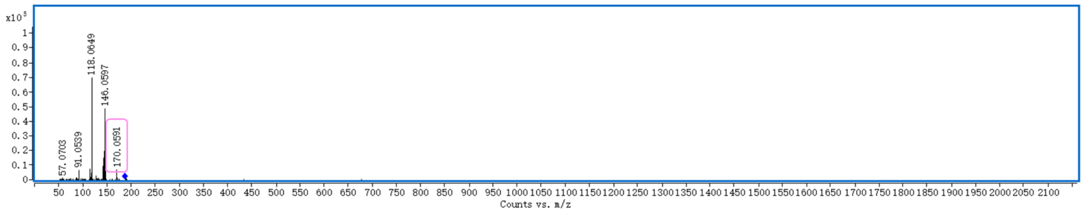

Compound 60

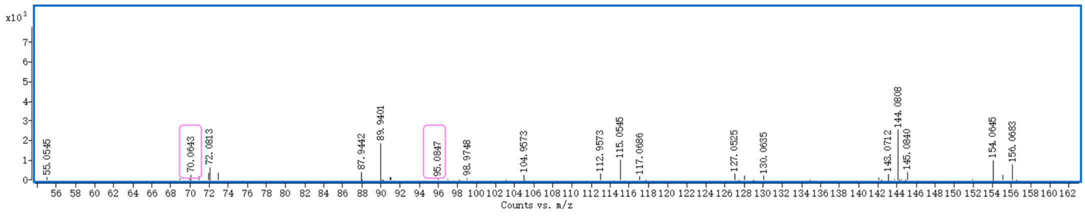

Compound 61

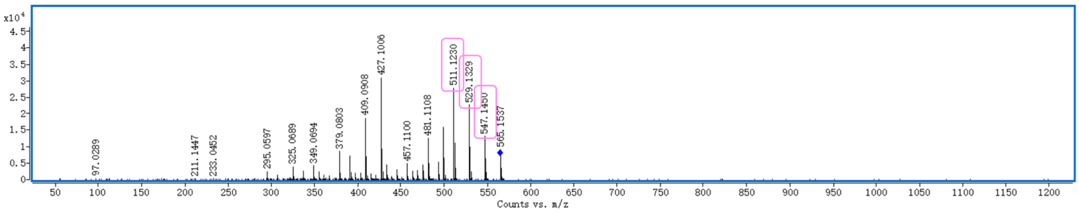

Compound 62

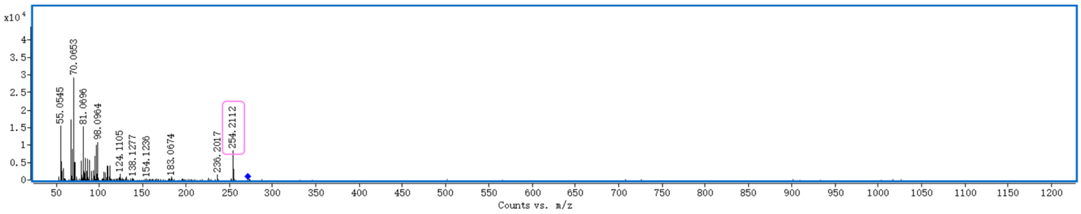

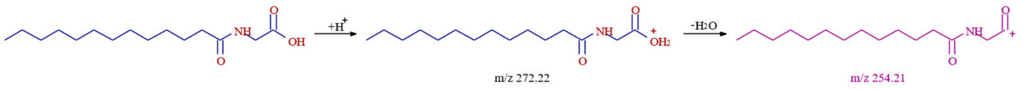

Compound 63

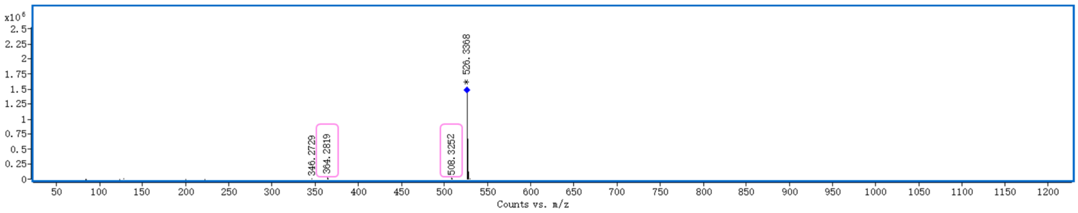

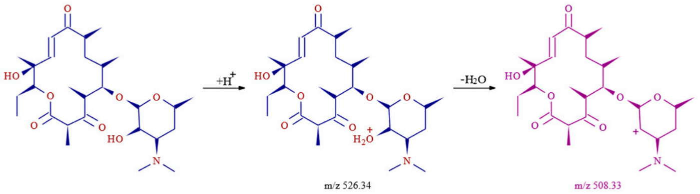

Compound 64

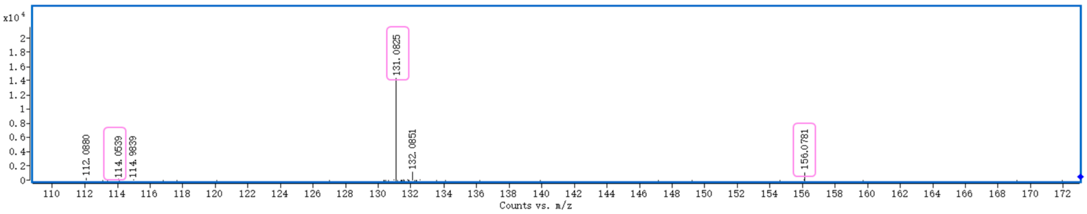

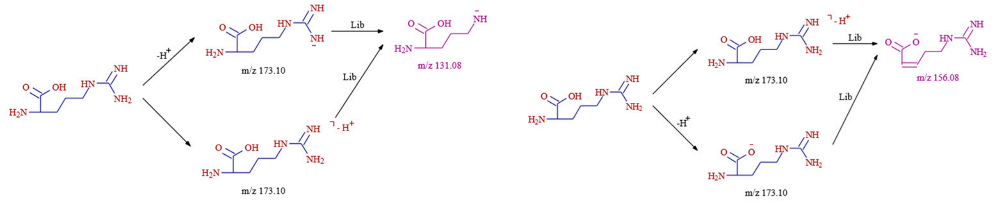

Compound 65

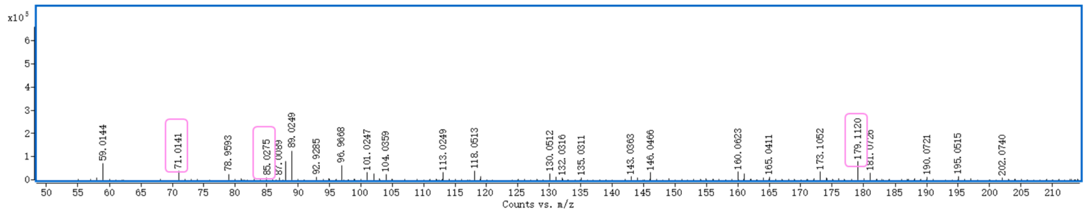

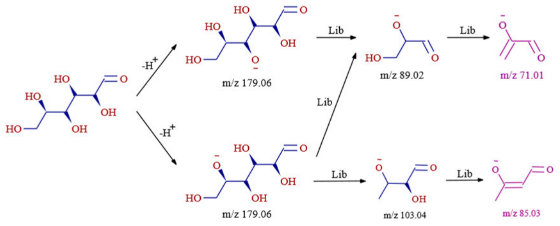

Compound 66

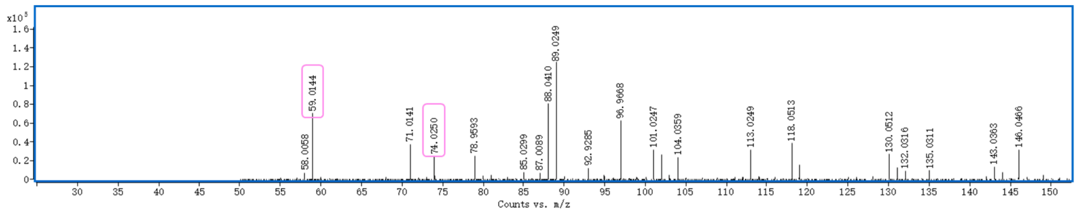

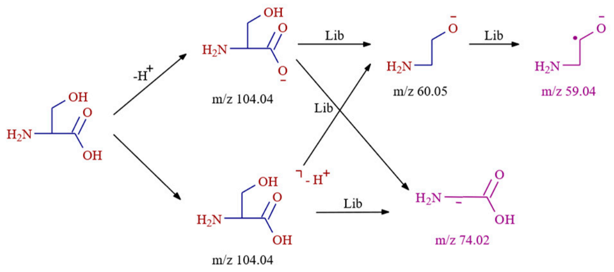

Compound 67

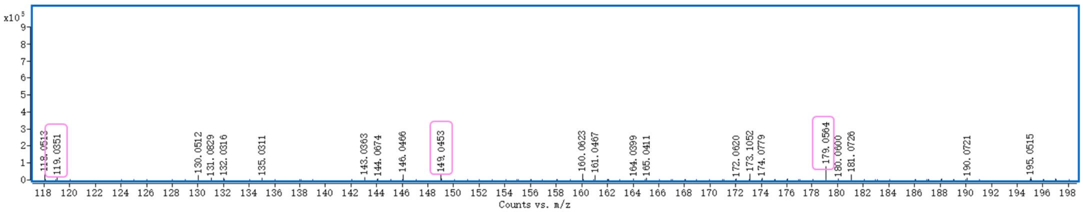

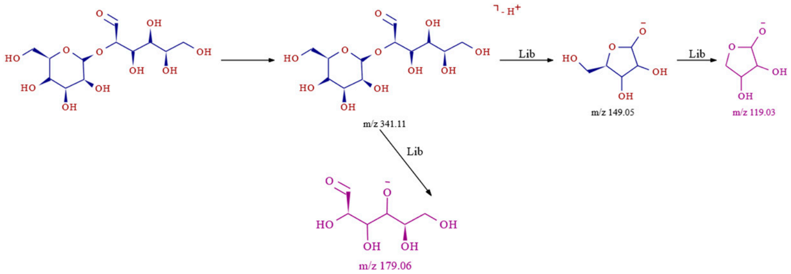

Compound 68

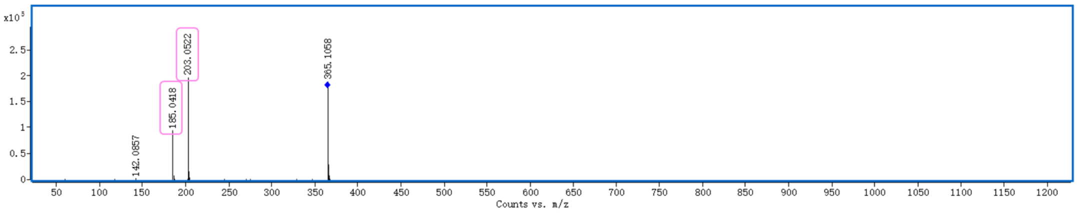

Compound 69

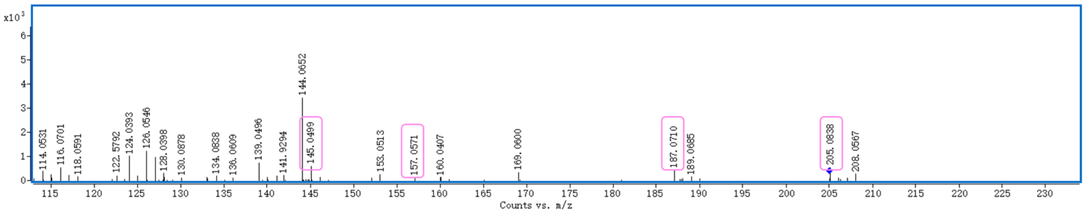

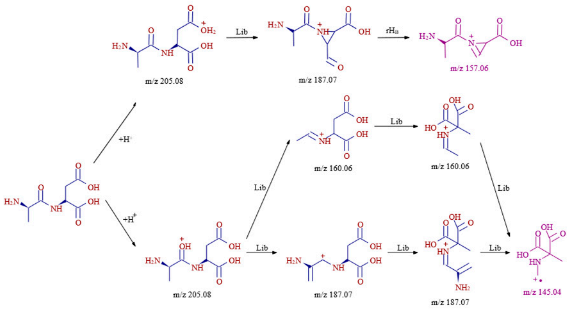

Compound 70

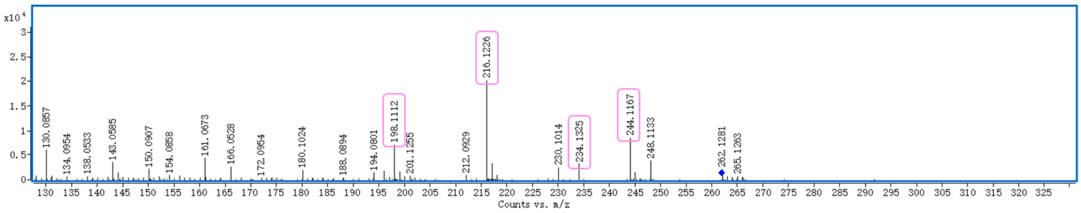

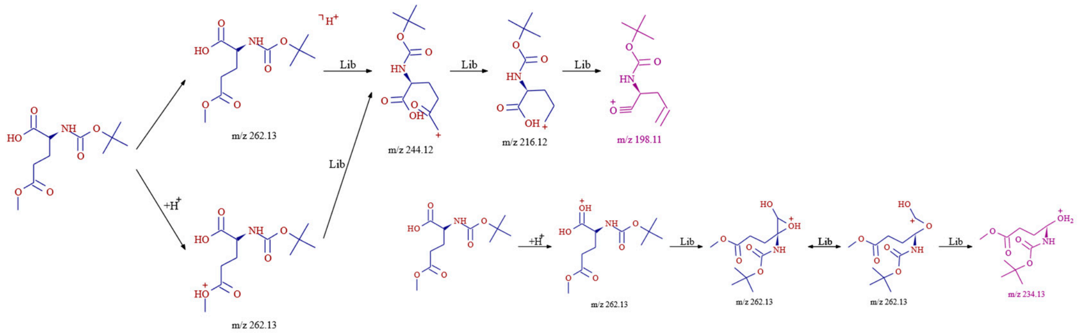

Compound 71

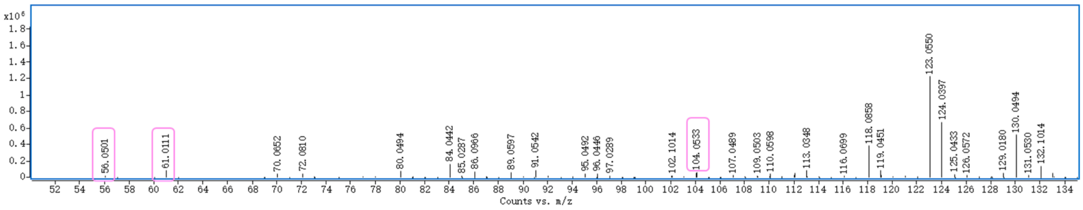

Compound 72

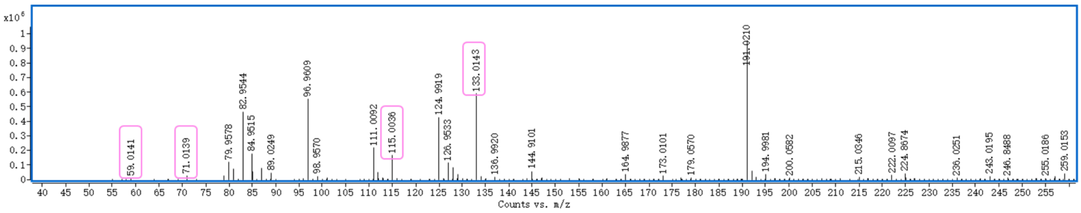

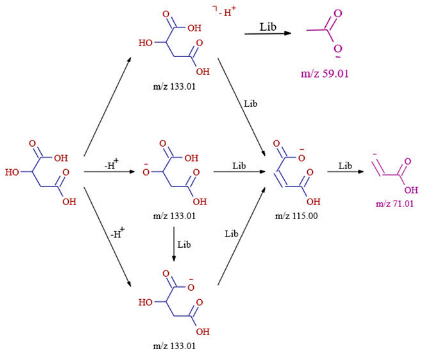

Compound 73

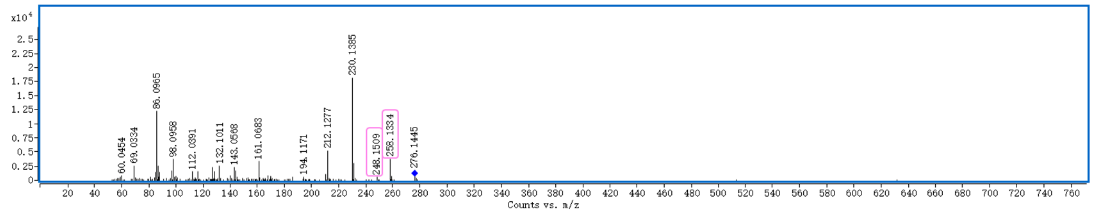

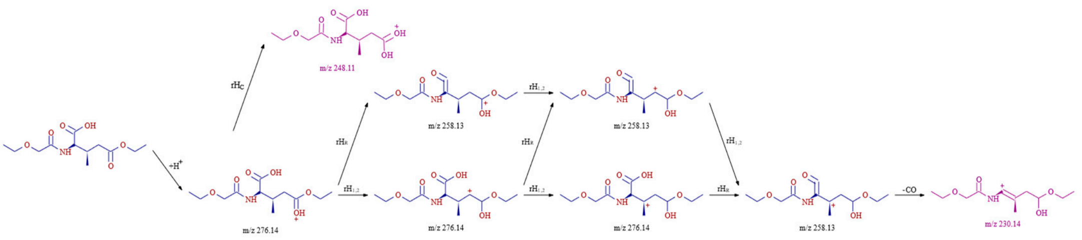

Compound 74

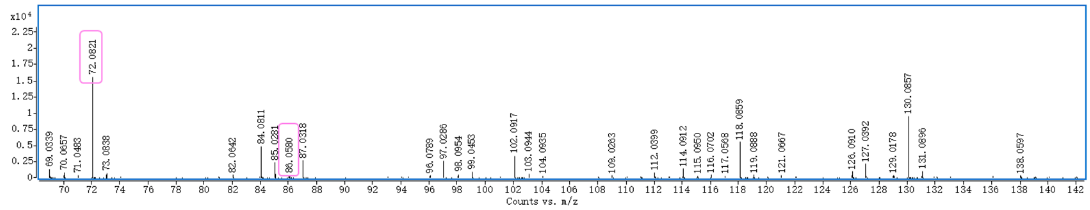

Compound 75

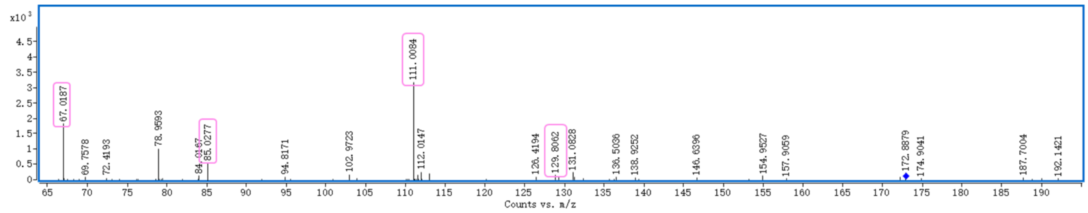

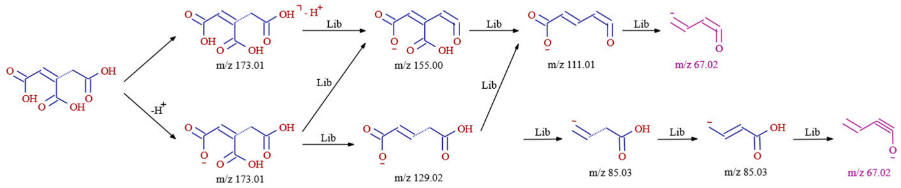

Compound 76

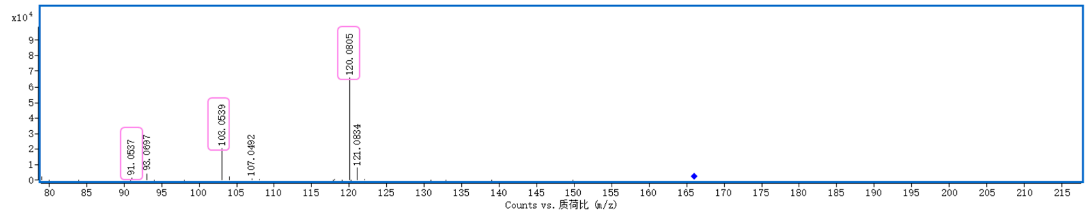

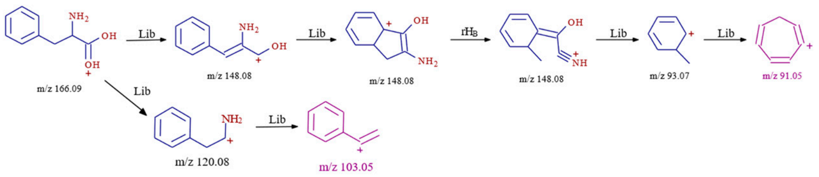

Compound 77

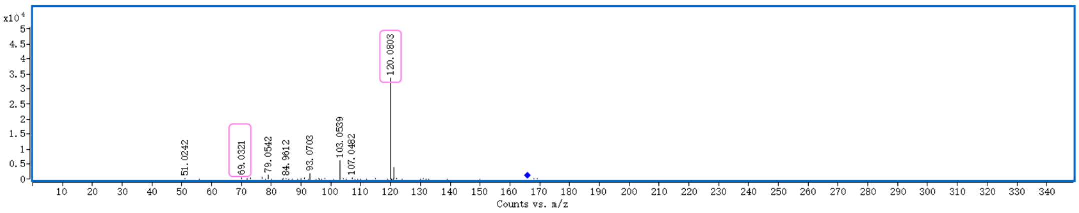

Compound 78

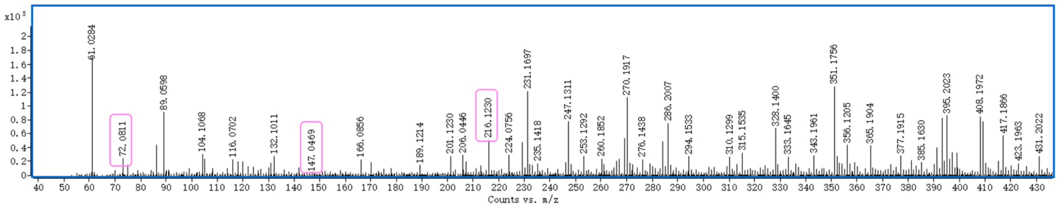

Compound 79

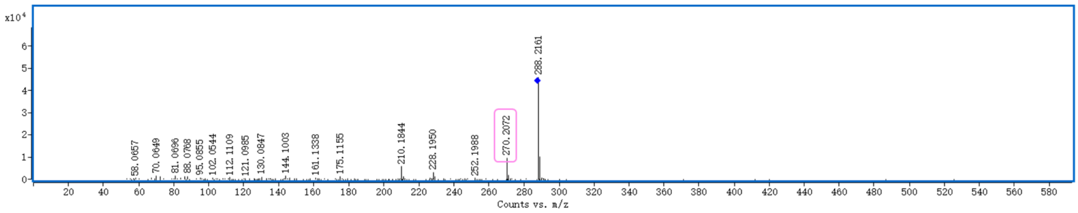

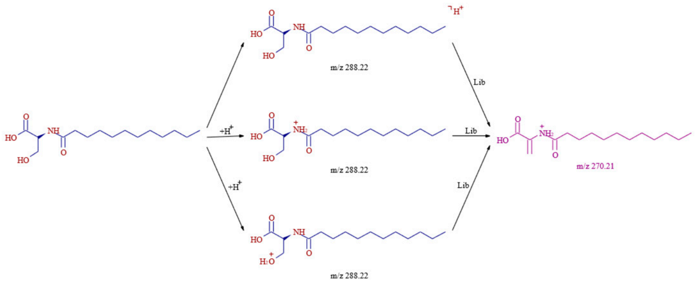

Supplement: Supplementary file 1 [file molecules-31-01322-s001.zip › molecules-4132529-supplementary.pdf]
